# Tracking *Pseudomonas aeruginosa* transmissions due to environmental contamination after discharge in ICUs using mathematical models

**DOI:** 10.1371/journal.pcbi.1006697

**Published:** 2019-08-28

**Authors:** Thi Mui Pham, Mirjam Kretzschmar, Xavier Bertrand, Martin Bootsma, on behalf of COMBACTE-MAGNET Consortium

**Affiliations:** 1 Julius Center for Health Sciences and Primary Care of the UMC Utrecht, Utrecht University, Utrecht, The Netherlands; 2 Centre for Infectious Disease Control, National Institute for Public Health and the Environment (RIVM), Utrecht, The Netherlands; 3 Hygiène Hospitalière, Centre Hospitalier Régional Universitaire, Besançon, France; 4 UMR 6249 Chrono-environnement, Université de Bourgogne-Franche-Comté, Besançon, France; 5 Department of Mathematics, Faculty of Sciences, Utrecht University, Utrecht, The Netherlands; University of Zurich, SWITZERLAND

## Abstract

*Pseudomonas aeruginosa* (*P. aeruginosa*) is an important cause of healthcare-associated infections, particularly in immunocompromised patients. Understanding how this multi-drug resistant pathogen is transmitted within intensive care units (ICUs) is crucial for devising and evaluating successful control strategies. While it is known that moist environments serve as natural reservoirs for *P. aeruginosa*, there is little quantitative evidence regarding the contribution of environmental contamination to its transmission within ICUs. Previous studies on other nosocomial pathogens rely on deploying specific values for environmental parameters derived from costly and laborious genotyping. Using solely longitudinal surveillance data, we estimated the relative importance of *P. aeruginosa* transmission routes by exploiting the fact that different routes cause different pattern of fluctuations in the prevalence. We developed a mathematical model including background transmission, cross-transmission and environmental contamination. Patients contribute to a pool of pathogens by shedding bacteria to the environment. Natural decay and cleaning of the environment lead to a reduction of that pool. By assigning the bacterial load shed during an ICU stay to cross-transmission, we were able to disentangle environmental contamination during and after a patient’s stay. Based on a data-augmented Markov Chain Monte Carlo method the relative importance of the considered acquisition routes is determined for two ICUs of the University hospital in Besançon (France). We used information about the admission and discharge days, screening days and screening results of the ICU patients. Both background and cross-transmission play a significant role in the transmission process in both ICUs. In contrast, only about 1% of the total transmissions were due to environmental contamination after discharge. Based on longitudinal surveillance data, we conclude that cleaning improvement of the environment after discharge might have only a limited impact regarding the prevention of *P.A.* infections in the two considered ICUs of the University hospital in Besançon. Our model was developed for *P. aeruginosa* but can be easily applied to other pathogens as well.

## Author summary

Understanding the transmission dynamics of multi-drug resistant pathogens in intensive-care units is essential for designing successful infection control strategies. We developed a method that estimates the relative importance of several transmission routes of *Pseudomonas aeruginosa* (*P. aeruginosa*), a bacterium intrinsically resistant to multiple antibiotics and known to be a major contributor to hospital-acquired infections. Our model includes three different routes: background transmission, cross-transmission and environmental contamination. Since moist environments may serve as natural reservoirs for *P. aeruginosa*, we focused our study on environmental contamination. Patients contribute to a pool of pathogens by shedding bacteria to the environment. Natural decay and cleaning of the environment lead to a reduction of that pool. By assigning the bacterial load shed during an ICU stay to cross-transmission, we disentangled environmental contamination during and after a patient’s stay. Previous studies were able to assess the role of environmental contamination for specific hospitals using laborious and costly genotyping methods. In contrast, we only used surveillance screening data to estimate the relative contributions of the considered transmission routes. Our results can be used to tailor or assess the effect of interventions. Our model was developed for *P. aeruginosa* but can be easily applied to other pathogens as well.

## Introduction

Hospital-acquired infections are a major cause of morbidity and mortality worldwide [1]. In industrialized countries, about 5–10% of admitted acute-care patients are affected whereas the risk is even higher in developing countries [2].

Due to its intrinsic resistance to multiple antibiotics, *Pseudomonas aeruginosa* (short *P. aeruginosa* or *P. A.*) is an important contributor to nosocomial infections [3–5]. The most serious *P. aeruginosa* infections lead to bacteremia, pneumonia, urosepsis, wound infection as well as secondary infection of burns [6]. In 2018, the World Health Organization has recognized *P. aeruginosa* as a serious health-care threat by including it in the list of antibiotic-resistant highest priority pathogens [7].

Given the severe consequences of *P. aeruginosa* infections, in particular for critically-ill patients, it is clear that strategies preventing infections are seen as a key priority. However, infections are recognized as only the tip of the iceberg, while colonizations represent the true load of pathogens carried by patients in the intensive-care unit (ICU). Understanding the dynamics of *P. aeruginosa* colonizations is therefore crucial for developing and evaluating infection control policies.

There are several modes of transmission for colonizations. An overview of the reservoirs and modes of *P. aeruginosa* transmission can be found, e. g. in [8]. Potential sources of colonization can be categorized into those with endogenous and exogenous origin. Colonization from endogenous sources is due to e. g. antibiotic selection pressure and was regarded as the most important route of *P. aeruginosa* [9–13]. However, more and more evidence has emerged on the importance of exogenous sources: Cross-transmission usually caused by temporarily contaminated hands of health-care workers (HCWs) has been identified as an additional source of transmission [14–19]. It is furthermore known that moist environments (e. g. soil and water) may serve as natural reservoirs of *P. aeruginosa* and that it can persist for months on dry inanimate surfaces [20]. Several studies have been performed to asses the sources of environmental contamination leading to cross-colonization. A rapid systematic review is given by [21].

Quantifying the relative importance of routes of transmission may serve as an essential tool in designing effective and tailored control strategies. There is little quantitative evidence in the scientific literature regarding the relative contribution of environmental contamination within the transmission dynamics of *P. aeruginosa* especially for non-epidemic situations. Prior investigations for *P. aeruginosa* are molecular epidemiological rather than modeling studies. Others have been modeling the importance of contaminated surfaces on the transmission of other nosocomial pathogens, e. g., for Methicillin-resistant Staphylococcus aureus (MRSA) and Vancomycin-Resistant Enterococci (VRE) [22–26]. However, they rely on deploying specific values for model parameters corresponding to the environment. Such information was obtained from previous studies that conducted extensive epidemiological surveillance in combination with costly, laborious as well as time-consuming methods of genotyping. Thus, these methods cannot be easily applied to other nosocomial pathogens without this cumbersome preliminary work. Therefore, an important question emerged: Can we quantify the impact of environmental contamination of *P. aeruginosa* on the transmissions within ICUs after the discharge of patients, using only longitudinal data?

In this paper, we present a mathematical transmission model that differentiates between three modes of transmission based only on longitudinal routine surveillance data. In particular, we are interested in estimating the relative contribution of environmental contamination after discharge. We used data from two ICUs of the University hospital in Besançon to estimate the parameters that characterize the transmission routes. The estimation procedure is based on a data-augmented Markov chain Monte Carlo simulation [27]. To our knowledge, this is the first quantitative analysis of the impact of environmental contamination after discharge on *P. aeruginosa* transmissions in ICUs using solely routine surveillance data.

## Materials and methods

In this section, we present our framework for modeling the transmission routes of *P. aeruginosa* including environmental contamination, as well as the method for computing the relative contributions of the routes. We further elaborate on the procedure that we used to estimate the relevant transmission parameters. A brief introduction to the data used for the analysis is given. We describe the model selection as well as model assessment procedures that are used to compare the developed models and to assess the model fit to the data.

### Transmission models

The underlying model for our algorithm is a SI-model (e.g. [28]). All patients are admitted to an ICU and either belong to the susceptible (*P. aeruginosa* negative) or colonized (*P. aeruginosa* positive) compartment at any given time. The latter includes patients with asymptotic carriage and those with *P. aeruginosa* infection.

A susceptible patient may become colonized at a certain transmission rate, which depends on the colonization pressure in the ward at the time. The corresponding transmission process is modeled by three different modes of transmission through which colonization can be acquired. They are distinguished based on the different patterns in the prevalence time series induced by each of them. *Background transmission* is independent of other patients and is represented as a constant rate. Sources may be antibiotic selection pressure as well as the introduction by visitors or permanently contaminated environments, such as sinks or air-conditioning. Consequently, this route comprises endogenous and exogenous sources that lead to a prevalence which fluctuates around the mean value. The corresponding probability of acquisition for an uncolonized patient is therefore assumed to be constant during the time period. *Cross-transmission*, usually occurring via temporarily contaminated hands of health-care workers, is proportional to the fraction of colonized patients in the wards. The probability of colonization due to cross-transmission is high if the number of colonized patients is high and vice versa. *Environmental contamination* is modeled on a ward-level represented as a general pool of bacteria linked to objects contaminated by colonized patients. We focus on the bacterial load that may persist in the environment even after the discharge of patients. This leads to higher probabilities of acquiring colonization after outbreaks, even when the number of colonized patients is low.

**Fig 1:**
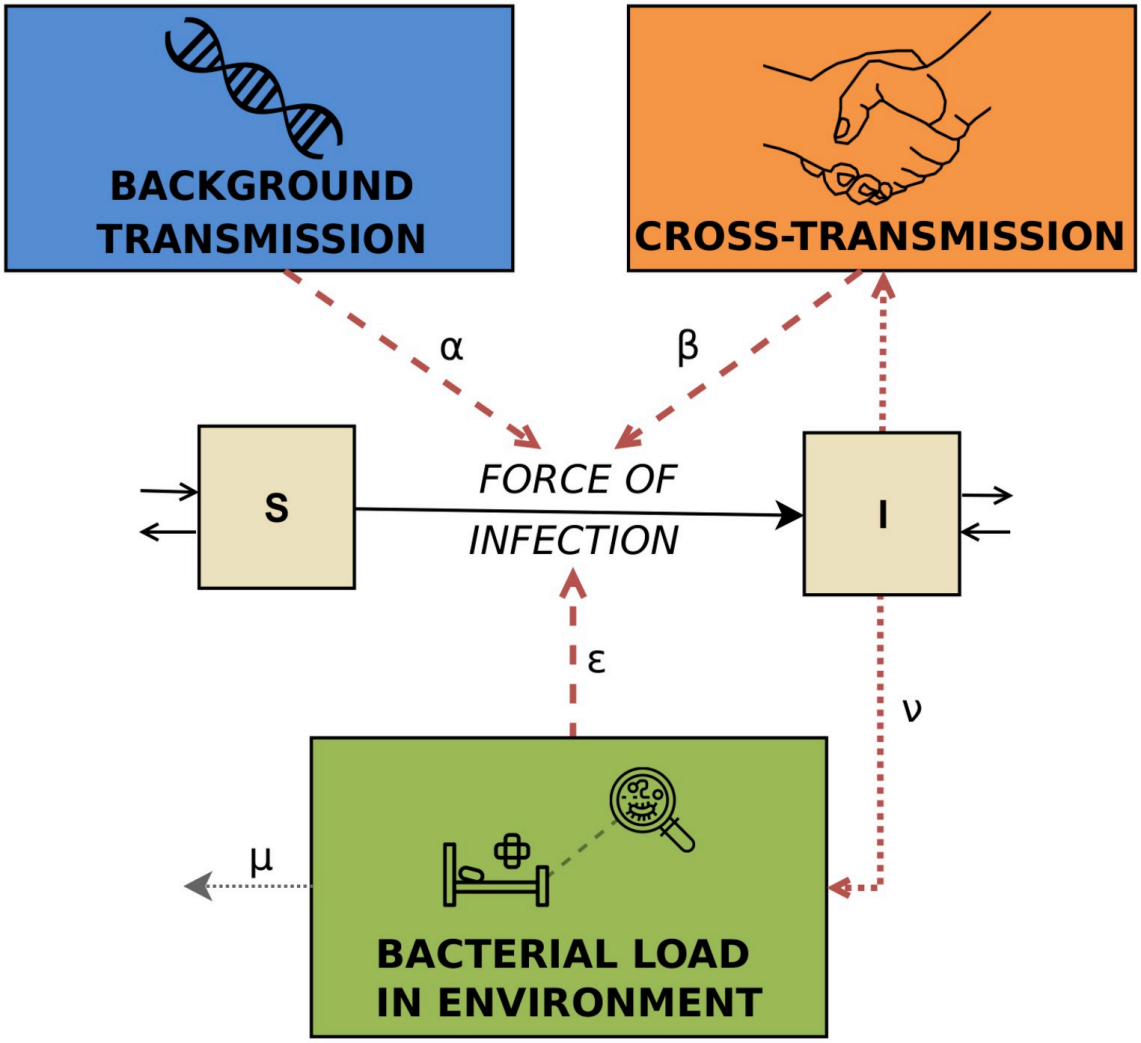
Schematic of the full transmission model. It represents the three different routes, i.e. background transmission, cross-transmission and environmental contamination.

The force of infection λ(*t*), i.e. the probability per unit of time *t* for a susceptible patient to become colonized, is modeled as

$$\begin{array}{c} \lambda \left(t\right)=\alpha +\beta \frac{I(t)}{N(t)}+\epsilon E\left(t\right) \end{array}$$ (1)

where *I*(*t*) is the number of colonized patients, *N*(*t*) the total number of patients and *E*(*t*) is a compartment tracking the overall bacterial load present in the ward at time *t*. The parameters *α*, *β* and *ϵ* are transmission parameters linked to the background transmission term, fraction of colonized patients and the environmental bacterial load, respectively. Under the assumption of a force of infection λ(*x*) at time *x*, the cumulative probability of any given susceptible person of becoming colonized in [0, *t*] is $1-e^{-\int_{0}^{t}\lambda \left(x\right)dx}$ (see e.g. [29]). A schematic of the transmission model is presented in Fig 1.

The described model is subject to the following further assumptions:

- Once colonized, patients remain colonized during the rest of the stay. This assumption is appropriate when the average length of stay of patients does not exceed the duration of colonization, as is the case for *P. aeruginosa*.
- Colonization is assumed to be undetectable until a certain detectable bacterial level is reached. We do not distinguish between several levels of colonization. Furthermore, the detection of carriage in specimen is assumed to be the same for each screening separately.
- Assuming that HCWs are contaminated for a short period of time (typically until the next disinfection) in comparison with the length of carriage for patients, we use a quasi-steady state approximation [28]. This means that contact patterns between patients and HCWs are not explicitly modeled and we assume direct patient-to-patient transmission.
- All strains of *P. aeruginosa* are assumed to have the same transmission characteristics. We therefore assume that all colonized patients may be a source of transmission and contribute equally to the colonization pressure.
- All susceptible patients are assumed to be equally susceptible.

In order to analyze the impact of environmental contamination after the discharge of colonized patients, we model the underlying mechanism leading to the presence of pathogens in the environment after discharge. Patients contribute to the overall bacterial load by shedding *P. aeruginosa* at a rate *ν* during their stay. Furthermore, natural clearance and cleaning lead to a reduction of *P. aeruginosa* bacteria in the environment at a rate *μ*. The change of environmental contamination can be described by

$$\begin{array}{c} \frac{dE}{dt}=\nu \frac{I(t)}{N(t)}-\mu E\left(t\right). \end{array}$$ (2)

The differential Eq (2) is solved by assuming *I*(*t*) = *I*_*t*_ and *N*(*t*) = *N*_*t*_ are known piece-wise constant functions with steps at times *t*_0_, *t*_1_, …, *t*_*N*_. Solving (2) using separation of variables leads to the overall bacterial load in the ward at time *t*:

$$\begin{array}{c} E\left(t_{i}\right)=E_{t_{i-1}}e^{-\mu (t_{i}-t_{i-1})}+\frac{\nu }{\mu }\frac{I_{t_{i-1}}}{N_{t_{i-1}}}(1-e^{-\mu (t_{i}-t_{i-1})}) \end{array}$$ (3)

for *t*_*i*_ ∈ {*t*_0_, …, *t*_*N*_} and

$$\begin{array}{c} E\left(t\right)=E_{\left\lfloor t\right\rfloor }e^{-\mu (t-\lfloor t\rfloor )}+\frac{\nu }{\mu }\frac{I_{\left\lfloor t\right\rfloor }}{N_{\left\lfloor t\right\rfloor }}(1-e^{-\mu (t-\lfloor t\rfloor )}) \end{array}$$ (4)

for ⌊*t*⌋ ≔ max{*x* ∈ {*t*_0_, …, *t*_*N*_}|*x* ≤ *t*} and $t\in R\backslash \{t_{0},t_{1},\ldots ,t_{N}\}$. The initial amount of bacterial load is denoted by *E*_0_ ≔ *E*(*t*_0_). The full details of deriving Eqs (3) and (4) from (2) are given in S1 Text.

Given the number of colonized patients at a certain time *t*, the bacterial load *E*(*t*) is deterministic. The acquisitions are stochastic based on the force of infection in (1). Our developed transmission model is therefore a hybrid of a stochastic and deterministic model.

All parameters, namely *α*, *β*, *ϵ*, *μ*, *ν* and *E*_0_ are assumed to be non-negative. By setting certain transmission parameters (*α*, *β* or *ϵ*) to zero, model variants may be defined. In this paper, we additionally consider a submodel with *ϵ* = 0, where environmental contamination is not explicitly modeled and therefore only two transmission routes are considered. The force of infection for this transmission model with two acquisition routes is then given by $\lambda \left(t\right)=\alpha +\beta \frac{I(t)}{N(t)}$.

### Relative contributions of transmission routes

For the prevention of colonization or infection with *P. aeruginosa*, specific intervention control strategies can be designed dependent on the relative importance of the transmission routes. However, for each observed acquisition of colonization, the responsible transmission route is unknown. And yet, for every acquisition, the probability that the colonization was due to a certain route can be estimated given that parameter values, the level of environmental contamination and the number of colonized patients are known. Thus, by estimating the transmission parameters *α*, *β*, *ϵ*, *μ* and *ν*, we were able to approximate the relative contributions of each transmission route to the total number of acquisitions.

The probability of acquisition can be approximated by the force of infection. It consists of different terms that can be assigned to the transmission routes under consideration, i.e.

$$\begin{array}{c} \lambda \left(t\right)=\lambda_{\text{background}}\left(t\right)+\lambda_{\text{cross-transmission}}\left(t\right)+\lambda_{\text{environment}}\left(t\right) \end{array}$$

**Fig 2:**
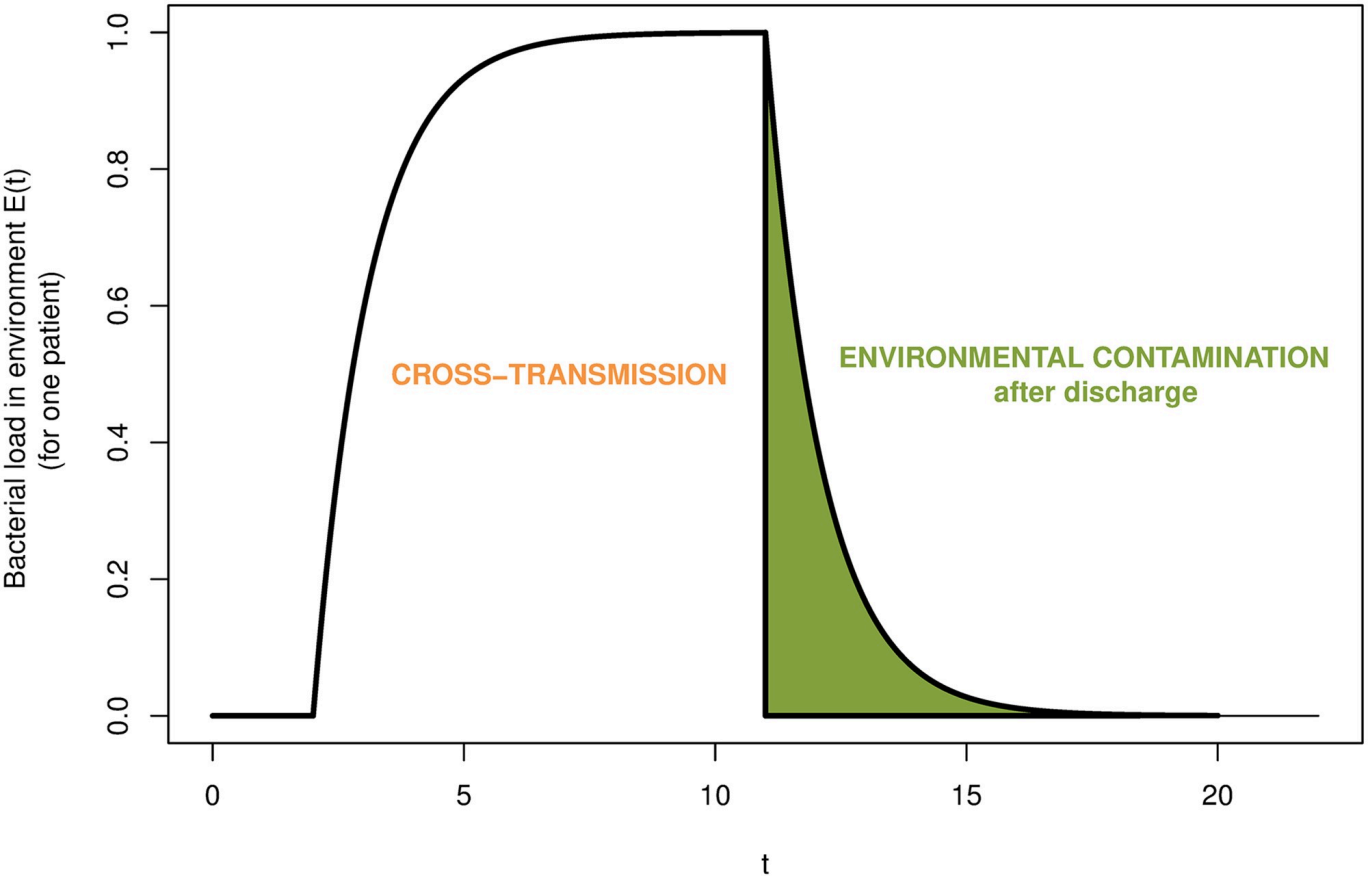
Schematic of the bacterial load shed by a patient developing over time. The bacterial load that is shed during a patient’s stay is assigned to cross-transmission. Environmental contamination after discharge accounts only for the bacterial load persisting after the discharge of that patient.

The primary aim of this paper is to estimate the relative contribution of environmental contamination after discharge in order to estimate the role of terminal environmental cleaning among ICU patients. According to our full model, bacterial load is produced by a colonized patient currently present. The cumulative bacterial load increases over time until the respective patient is discharged. After discharge, shedding of that particular patient stops and decreases over time. The bacterial load shed during a patient’s stay (which may then be transmitted via HCWs to other patients) is assigned to cross-transmission as in practice, it may not be distinguished from the classical definition of cross-transmission. The bacterial load persisting after discharge is the variable of interest and represents the impact of already discharged patients on the current transmissions in the ICU. A schematic of the bacterial load of a single patient over time and its attribution to the different transmission routes is visualized in Fig 2.

The previous explanation leads to the following attribution of the terms to the different acquisition routes

$$\begin{array}{cc} \lambda \left(t\right) & =\alpha +\beta \cdot \frac{I\left(t\right)}{N\left(t\right)}+\epsilon \cdot E\left(t\right)\\ & =\underset{\lambda_{\text{background}}}{\underset{︸}{\alpha }}+\underset{\lambda_{\text{cross-transmission}}\left(t\right)}{\underset{︸}{\left[\beta \cdot \frac{I\left(t\right)}{N\left(t\right)}+\epsilon \sum_{i_{p}}\frac{E_{i_{p}}\left(t\right)}{N\left(t\right)}\right]}}+\underset{\lambda_{\text{environment}}\left(t\right)}{\underset{︸}{\epsilon \left[\sum_{i_{d}}\frac{E_{i_{d}}\left(t\right)}{N\left(t\right)}+E_{0}e^{-\mu t}\right]}} \end{array}$$ (5)

where *i*_*p*_ indicates a colonized patient that is present at time *t* and *i*_*d*_ a colonized patient that has been colonized prior to *t* but was already discharged. The bacterial load produced by patient *i* at time *t* is given by

$$\begin{array}{c} E_{i}\left(t\right)=\{\begin{array}{cc} 0 & \text{for}\;t<t_{i}^{c}\\ \frac{\nu }{\mu }(1-e^{-\mu (t-t_{i}^{c})}) & \text{for}\;t_{i}^{c}\leq t<t_{i}^{d}\\ E_{i}\left(t_{i}^{d}\right)e^{-\mu (t-t_{i}^{c})} & \text{for}\;t\geq t_{i}^{d} \end{array} \end{array}$$

where $t_{i}^{c}$ is the time of colonization and $t_{i}^{d}$ the time of discharge of patient *i*.

In continuous time, the relative contribution of a specific route to the overall number of acquired colonizations is determined by the ratio of the probability of colonization due to that route and the probability of colonization:

$$\begin{array}{cc} \text{Contribution}\;\text{of}\;\text{route}\;j=R_{j} & =\frac{\sum_{i=1}^{l}\frac{P(\text{colonization}\;\text{at}\;\text{time}\;t_{i}^{c}\;\text{due}\;\text{to}\;\text{route}\;j)}{P(\text{colonization}\;\text{at}\;\text{time}\;t_{i}^{c})}}{\text{Number}\;\text{of}\;\text{acquisitions}}\\ & =\frac{\sum_{i=1}^{l}\frac{\lambda_{j}\left(t_{i}^{c}\right)}{\lambda (t_{i}^{c})}}{\text{Number}\;\text{of}\;\text{acquisitions}} \end{array}$$ (6)

where *l* is the number of colonized patients, $t_{1}^{c},\ldots ,t_{l}^{c}$ represent the times of colonization and *j* can be either of the three considered routes. The relative contributions are then given by:

- Contribution of background transmission $=R_{\text{background}}=\frac{\sum_{i=1}^{l}\frac{\alpha }{\lambda (t_{i}^{c})}}{\text{Number}\;\text{of}\;\text{acquisitions}}$
- Contribution of cross-transmission $=R_{\text{crossT}}=\frac{\sum_{i=1}^{l}\frac{\beta \cdot \frac{I(t_{i}^{c})}{N(t_{i}^{c})}+\epsilon \sum_{i_{p}}\frac{E_{i_{p}}\left(t_{i}^{c}\right)}{N(t_{i}^{c})}}{\lambda (t_{i}^{c})}}{\text{Number}\;\text{of}\;\text{acquisitions}}$
- Contribution of environmental contamination $=R_{\text{env}}=\frac{\sum_{i=1}^{l}\frac{\epsilon [\sum_{i_{d}}\frac{E_{i_{d}}\left(t_{i}^{c}\right)}{N(t_{i}^{c})}+E_{0}e^{-\mu t_{i}^{c}}]}{\lambda (t_{i}^{c})}}{\text{Number}\;\text{of}\;\text{acquisitions}}$

For the submodel including only background and cross-transmission, the computation of the relative contribution is derived from above by setting *ϵ* = 0.

More details on the calculations can be found in S3 Text. In practice, colonization events are observed only in discrete times. The formulas for the transmission model and the relative contribution are adapted for this discrete time assumption and are elaborated in S2 and S3 Texts. Since the calculations for the relative contributions of the transmission routes in the discrete-time scenario require the use of the gamma function and therefore become computationally intensive, we use the continuous-time formulas as approximations. Since values of the force of infection λ(*t*) are typically small (< 0.25), the force of infection itself is a good approximation of the probability of infection as $1-e^{-\int_{0}^{t}\lambda \left(x\right)dx}\approx \lambda \left(t\right)$ for small values of λ(*t*). Hence, the discrete-time formulas for the relative contributions can be approximated by the continuous-time formulas evaluated at discrete time steps.

### Estimation procedure

We assume that a patient is admitted to the ICU at time $t_{i}^{a}$ and discharged at time $t_{i}^{d}$. The probability that a patient is admitted already colonized is described by the importation probability *f*. The rate at which a susceptible patient transitions to being colonized is given by Eq (1). The colonization state of an individual patient is determined from screening information. We suppose that for each patient *i* a set of screening results $X_{i}=(X_{i}^{\left(1\right)},\ldots ,X_{i}^{\left(m\right)})$, taken on days $t_{i}^{\left(1\right)},\ldots ,t_{i}^{\left(m\right)}$ is available. The set of all screening results is denoted by *X* = {*X*_1_, …, *X*_*n*_} where *n* is the total number of patients. Since screening tests are typically intermittent and imperfect, we define the test sensitivity *ϕ*, i.e. probability that a colonized patient has a positive result.

The aim is to estimate the model parameters *α*, *β*, *ϵ*, *μ*, *ν* and *E*_0_ as well as the sensitivity of the screening test *ϕ* and the importation rate *f* based on longitudinal data. The relative contributions of the transmission routes can then be estimated following the description in (6). The key idea of the estimation procedure is to fit a stochastic transmission model to the observed data. It is based on certain patterns of fluctuations in the prevalence linked to the different transmission routes (as previously described in section *Transmission models*).

In the analysis, we use the following input data for each patient:

- day of admission
- day of discharge
- screening days and results.

Thus, we use a day as the smallest time unit in our model and assume that events occur in daily intervals. In principle, other time units may be chosen for an analysis if the required information on admission, discharge and culturing is available. However, smaller units may increase the computational time.

If transmission dynamics were perfectly observed, it would be straightforward to calculate the likelihood of the data given parameters *θ* = {*α*, *β*, *ϵ*, *μ*, *ν*, *ϕ*, *f*}. However, the true colonization time of a patient is typically unobserved which leads to uncertainty about the true prevalence at any given time. Hence, the likelihood is analytically intractable. The method developed by [27] overcomes this problem by augmenting the parameter space with the unobserved colonization times and sampling over this space using an Markov-chain Monte Carlo (MCMC) algorithm. We adapted this method for our purposes to estimate the posterior distributions of the model parameters. The joint likelihood is determined using three models: an observation model, a transmission and importation model, and a prior model. The observation model describes the imperfect observation of the transmission dynamics for given the (augmented) colonization times. The transmission and importation model describe the probabilities of the realizations given the model parameters. The prior model determines the distribution of the parameters a priori. The augmented data consists of a set of colonization statuses and times as well as importation markers. At each iteration, imperfectly observed colonization times are imputed and model parameters *θ* sampled that are consistent with the observed culture data. This approach accounts for imperfect and infrequent screening, missing admission and discharge swabs and leads to an estimation of the true (rather than the observed) prevalence on admission. Precise details of the analysis can be found in S5 Text. The algorithm was implemented in C++ and was tested using simulated data. Convergence of the MCMC chains were verified using visual inspection.

We used uninformative exponential priors Exp(0.001) for the transmission parameters *α*, *β*, *ϵ* and *μ*. Parameters for the proposal distribution were tuned in order to ensure rapid convergence. Similar to [30], we estimated the sensitivity *ϕ* and importation parameter *f* using uninformative beta prior distributions Beta(1, 1). The initial bacterial load *E*_0_ was approximated by $\frac{\nu }{\mu }\bar{I}$ with $\bar{I}$ being the mean prevalence in the ward. A discussion concerning the choice of parameters for the prior distribution is left to the supplementary section S8 Text.

The MCMC algorithm was run for 500, 000 iterations following a burn-in of 30, 000 iterations. The MCMC iterations were then thinned by a factor of 10, leaving 50, 000 iterations for inference. In each iteration, 20 data-augmentation steps were performed with each augmentation chosen at random. The algorithm was implemented in C++ and the analysis of the output was performed in R (Version 3.5.1) [31].

During the estimation process, several assumptions are made.

- Incorporating both sensitivity and specificity parameters in a model may cause identifiability issues. Thus, test specificity was assumed to be 100%, meaning that positive results were assumed to be true positive. Experimental results indicate the specificity of screening tests to be close to 100% [32].
- The initial bacterial load *E*_0_ is assumed to be the environmental contamination at the beginning of the study period. The effect of *E*_0_ diminishes proportionally to exp(−*μ*) per day. It is therefore sufficient to use an approximation rather than including it as a parameter in the estimation process. We use the equilibrium state of (2) as an approximation, i.e.
  $$\begin{array}{c} E_{0}\approx \frac{\nu }{\mu }\bar{I} \end{array}$$
  where $\bar{I}$ represents the mean prevalence in the ward.
- The environmental contribution to the force of infection at time *t* is *ϵ* ⋅ *E*(*t*). As the total amount of environmental contamination *E*(*t*) is unobserved, it is only possible to estimate the product *ϵ* ⋅ *E*(*t*). For *t* = 0, 1, 2, … it holds
  $$\begin{array}{c} \epsilon \cdot E\left(t\right)=\epsilon E_{0}e^{-\mu t}+\frac{\epsilon \cdot \nu }{\mu }\sum_{i=0}^{t-1}\frac{I_{i}}{N_{i}}\left(1-e^{-\mu }\right){\left(e^{-\mu }\right)}^{t-1-i}. \end{array}$$
  The parameter *ν* is always integrated in the product *ϵ* ⋅ *ν*. Hence, instead of estimating *ϵ* and *ν* separately, it is sufficient to estimate the product *ϵ* ⋅ *ν*.
- Colonization was defined as the presence of bacteria at the screening sites as reported in the available data. Admission and screening are assumed to occur at 12:00 pm and discharge at 11:59 am.
- Re-admissions are not accounted for. Instead every new admission is treated as a new patient. The probability to be positive on admission is therefore identical for all patients, irrespective whether it is a readmission or not. Since we are interested in the overall prevalence and overall relative contribution of the acquisition routes rather than individual predictions, we do not expect this to have a major influence on our results.
- Since the smallest time unit is one day, colonization events occurring on a particular day are assumed to be independent.
- A negative result on the day of colonization is considered to be a false negative result.
- It is assumed that colonized patients contributed to the total colonized population from the day after colonization onwards, or for importations, from the day of admission. This assumption leads to an underestimation of the number of acquisitions for colonization times at the beginning of the day (but just after screening). On the other hand, since pathogenic bacteria such as P. aeruginosa undergo a *lag phase* during their growth cycle, in which the bacteria adapt to the new environment and are not yet able to divide, onward transmission events are likely to be rare during the early stages of colonization. Therefore, the number of onward transmissions are likely to be overestimated for colonizations occurring at the end of the day.

### Data

The data used in the current analysis were collected from two ICUs, denoted by A and B, between 1999 and 2016 at University Hospital of Besançon, eastern France, in the framework of a systematic screening for *P.aeruginosa*. The data sets include admission and discharge dates as well as dates, sites and results of culturing of adult patients. ICU A is a surgical ICU that comprised 15 beds in the time period 1999-2008 and 20 from 2010 till 2016. The ICU was renovated between 2008 and 2009 and the number of beds was increased after completion of the renovation work. ICU B, a non-surgical ICU, had 15 beds from 2000 till 2011 and increased to 20 beds afterwards. Rectal and nose swabs were obtained upon admission (during the first 48 hours) and once a week thereafter. A positive result on one of the swabs was counted as a positive culture. A negative culture resulted from a negative culture on both swabs taken at the specific day. More than 84% of admitted patients were screened. As HCWs, including physicians, were (with minor exceptions) working only in one of the ICUs during the whole study period, the two ICUs can be treated independently in the analysis.

Since 2000, the hand hygiene procedures recommended in both ICUs is rubbing with alcohol-based gels, or solutions (ABS). Cleaning of the rooms is done daily by using the detergent-disinfectant Aniosurf^®^. The sinks were cleaned daily before pouring the detergent-disinfectant Aniosurf^®^ into the U-bends. Plumbing fittings were descaled weekly.

In our main analysis, data for each ICU and each time period (before and after renovation) was treated as distinctive data sets, resulting in four different analyses. No pooling of the results were performed. In a second analysis, the data for the different time periods and different ICUs were combined. The results are compared with the main analysis and are presented in S1 and S2 Tables. Each data set was analyzed using

- the full model including background transmission, cross-transmission and environmental contamination after discharge,
- the submodel with only background and cross-transmission.

Patient data were anonymized and de-identified prior to analysis.

### Model selection

To assess the relative performance of a given model, we used a version of the deviance information criterion (DIC) based on [33]. For an estimated parameter set *θ* and observed data set *x* it is computed as the expected deviance plus the effective number of parameters: $\text{DIC}=\bar{D_{x}\left(\theta \right)}+p_{D}$. A lower value indicates a better fit. The effective number of parameters *p*_*D*_ represents a complexity measure and is calculated by the difference of the posterior mean deviance and the deviance at the posterior mean: $\bar{D_{x}\left(\theta \right)}-D_{x}\left(\tilde{\theta }\right)$. In this paper, we use the approximation $p_{D}=\frac{1}{2}\text{var}\left(D_{x}\left(\theta \right)\right)$ introduced by [33].

The DIC is a simple measure that can be used to compare hierarchical models. Furthermore, it allows determining whether two data sets may be concatenated or should be treated separate. The idea is to distinguish two models: one that includes one parameter set for both ICUs (and therefore treats them as concatenated) and one that includes different parameter sets for each ICU (and thus treats them as separate). The first scenario leads to one analysis and one DIC value whereas the second model results in two independent analyses and hence two DIC values. The sum of the DICs of the latter may be compared to the DIC value of the first scenario. A smaller DIC value is preferred. More details can be found in S6 Text.

### Model assessment

We chose to check the adequacy of the models using the following approach. The ability of the model to predict the probability of acquisition based on the predicted force of infection was assessed. The computed numerical values for the force of infection are assigned to a bin representing the segment covering the numerical value. For a given value λ of the force of infection, the theoretical probability of acquisition *p*_acq_ per susceptible patient is computed by 1 − exp(−λ). The predicted fraction of acquisitions *f*_acq_ is computed by dividing the number of acquisitions *N*_acq_ by the number of susceptible patients *N*_susc_. We compute 95% confidence intervals assuming that the number of acquisitions follows a binomial distribution of Bin(*N*_susc_, *f*_acq_). The described method is performed for 100 MCMC updates. Coverage probabilities are computed to determine the actual proportion of updates for which the interval contains the theoretical probability of acquisition. We set the nominal confidence level to 0.95. A good fit is given when the actual coverage probability is (more or less) equal to the nominal confidence level. In order to avoid the coverage probability tending to zero when *p*_acq_ tends to 0 or 1, Jeffreys confidence intervals are used (as recommended in [34]). When *N*_acq_ = 0 the lower limit is set to 0, and when *N*_acq_ = *N*_susc_ the upper limit is set to 1.

## Results

### Descriptive analysis of data

The descriptive statistics of the data sets corresponding to ICU A and B with respect to the number of admissions, lengths of stay and colonization characteristics are shown in Table 1. The time period referred to as *before renovation* (short before) is defined as 20/04/1999 − 23/01/2008 (approx. 8.8 years) for ICU A and 11/01/2000 − 12/01/2011 (approx. 11 years) for ICU B. The time period referred to as *after renovation* (short after) is defined as 31/05/2008 − 30/12/2016 (approx. 6.4 years) for ICU A and 13/01/2011 − 13/09/2016 (approx. 5.7 years) for ICU B. In ICU A, the number of beds decreased during the renovation. Hence, we decided to remove the renovation period from the analysis for ICU A.

**Table 1:** Descriptive statistics for the *P. aeruginosa* carriage data collected from two ICUs at University hospital Besançon, France, 1999-2016.

	No.			Median (IQR)*		%	
	Before ^†^	After ^‡^	Total	Before	After	Before	After
**ICU A**							
Study length, days	3200	2340	5540				
Readmissions	320	278	598			9.8	8.2
Admissions^§^	3261	3398	6659				
Length of stay, days				8.0 (3.0-19.0)	8.0 (3.0-16.0)		
Importations^¶^	50	87	137			1.5	2.6
Observed *P.A.* acquisitions^‖^	350	270	620			10.7	7.9
**ICU B**							
Study length, days	4020	2079	6099				
Readmissions	406	334	740			9.3	9.9
Admissions	4360	3384	7744				
Length of stay, days				8.0 (3.0-18.0)	8.0 (3.0-15.0)		
Importations	127	124	251			3.7	2.9
Observed *P.A.* acquisitions	504	395	899			11.6	11.7

* Interquartile range

^†^ 20/04/1999 − 23/01/2008 for ICU A and 11/01/2000 − 12/01/2011 ICU B.

^‡^ 31/05/2009 − 30/12/2016 for ICU A and 13/01/2011 − 13/09/2016 for ICU B.

^§^Each ICU stay was counted separately, even in case of a multiple ICU stay within a given hospitalization.

^¶^ Patients positive on admission; false negative results are not taken into account.

^‖^ An acquisition is when a patient test negative on admission and had a postive result before discharge; false negative results are not taken into account.

In total, 13,065 patients (6,061 admitted to ICU A and 7,004 to ICU B) and 37,738 screening results (14,631 in ICU A and 23,107 in ICU B) were included in the analysis. The number of readmissions is higher for ICU B than for ICU A. In our analysis, every admission was treated separately (as a new patient) resulting in 14,403 admissions (6,659 admitted to ICU A and 7,744 to ICU B).

The corresponding median length of stay was 8.0 days for both ICUs before and after renovation, respectively. Hence, there is hardly any difference between the ICUs, nor between the two time periods regarding the median length of stay.

**Fig 3:**
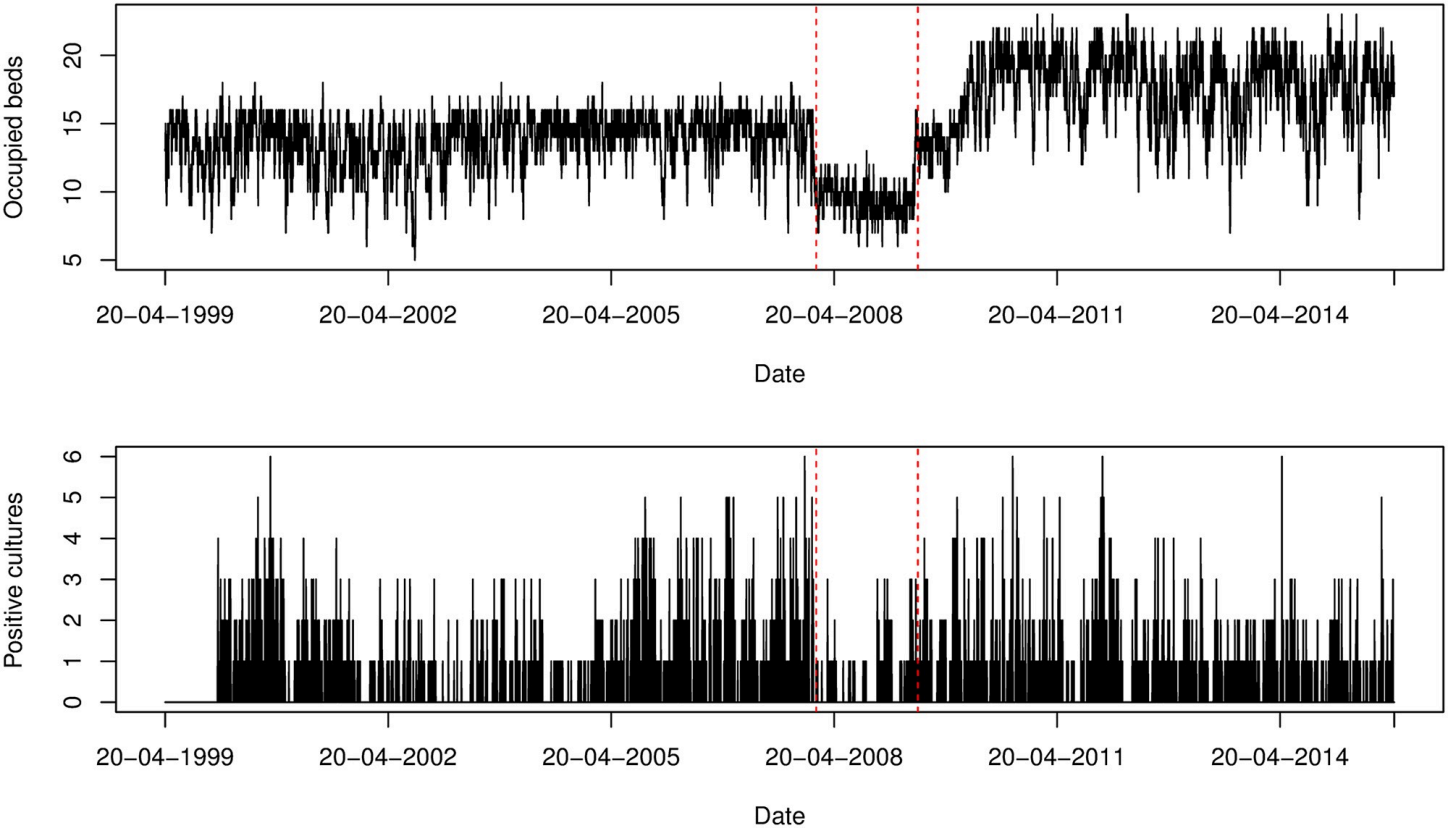
Number of occupied beds and positive isolates cultured from patients per swab day for ICU A. The red dotted lines indicate the time points that splits the study period into *before renovation* (20/04/1999 − 23/01/2008) and *after renovation* (31/05/2009 − 30/12/2016). Since the number of beds decreased during the renovation, the period is removed from the analysis.

**Fig 4:**
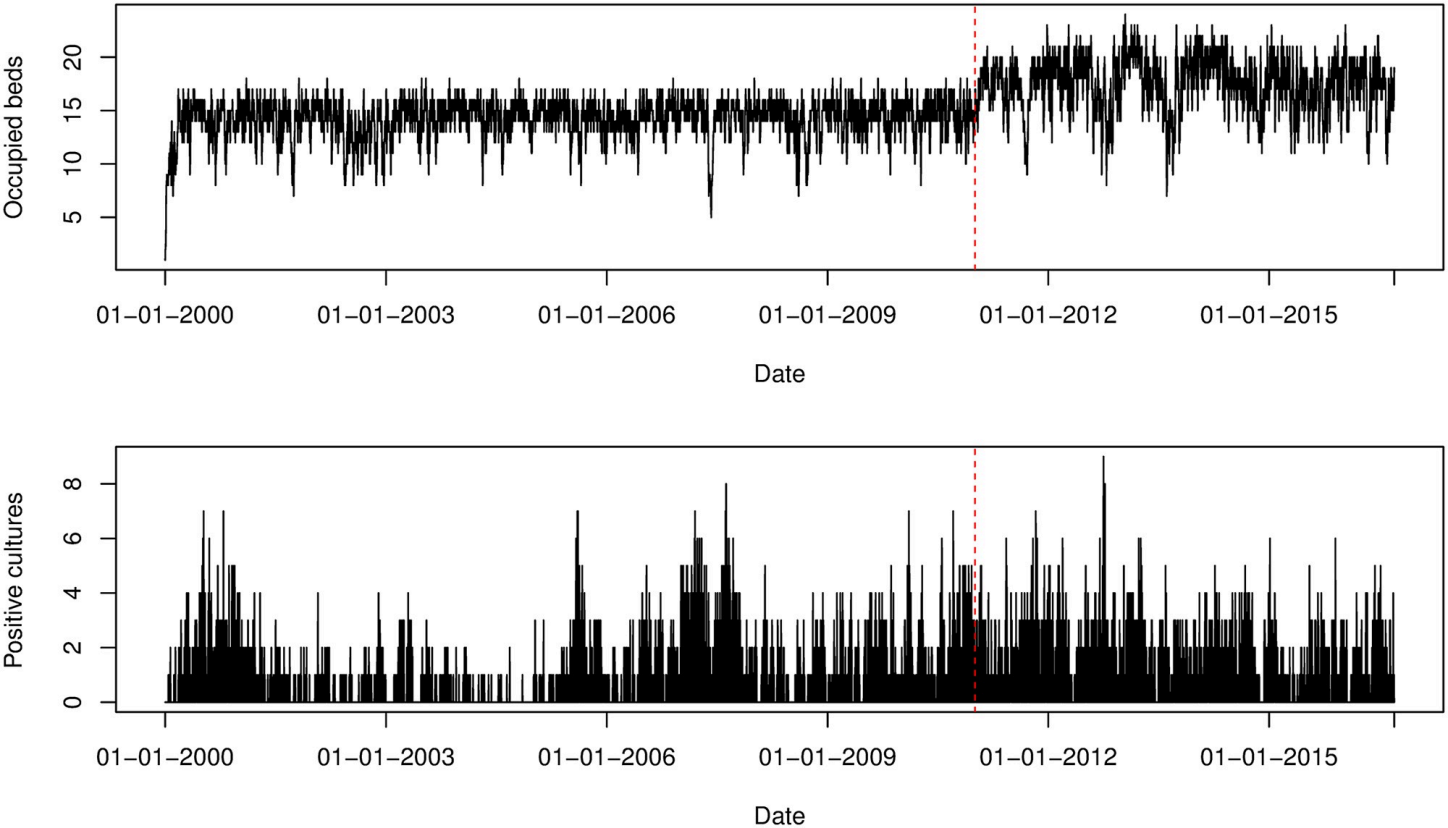
Number of occupied beds and positive isolates cultured from patients per swab day for ICU B. The red dotted line indicates the time point that splits the study period into *before renovation* (11/01/2000 − 12/01/2011) and *after renovation* (13/01/2011 − 13/09/2016).

The fraction of patients who were positive on admission was slightly higher after renovation in ICU A. The reverse is true for ICU B. The observed fraction of patients who acquired colonization slightly decreased after renovation in both ICUs. There were 1,519 patients (620 in ICU A and 899 in ICU B) observed to be colonized during their stay and 388 patients (137 in ICU A and 251 in ICU B) observed to be colonized on admission. The percentage of patients admitted positively on admission and with acquired colonization is higher in ICU B than in ICU A. The total number of patients per ICU and the number of positive cultures are visualized in Figs 3 and 4.

### Estimated model parameters

Two model variants were fitted to the Besançon ICU data aiming to estimate the set of parameters *θ*_1_ = {*α*, *β*, *ϕ*, *f*} and *θ*_2_ = {*α*, *β*, *ϵ*, *μ*, *ϕ*, *f*} corresponding to the submodel with only two and the full model with all three transmission routes, respectively.

#### Submodel: Two transmission routes

Posterior estimates of the model parameters for each ICU and each time period are reported in Table 2. Acceptance probabilities for proposed updates to the augmented data ranged from 3.2% (ICU B after renovation) to 11.1% (ICU A before renovation). Pairwise scatter plots indicated little correlation between parameter values, with the exception of a negative correlation between *α* and *β* (see S1 Fig). Histogram and trace plots of the posterior estimates are given in S2–S5 Figs and show that the MCMC chains rapidly mix and quickly converge to their stationary distribution. We found our estimates to be robust to the choice of priors for transmission parameters.

**Table 2:** Summary statistics of the marginal posterior distributions for parameters of the submodel based on the analysis of the Besançon data.

Parameter	Symbol	Median (95% credibility interval)*			
		ICU A		ICU B	
		Before ^†^	After ^‡^	Before	After
Background coefficient	*α*	0.011 (0.006, 0.016)	0.013 (0.009, 0.016)	0.007 (0.005, 0.01)	0.014 (0.009, 0.018)
Cross-transmission coefficient	*β*	0.043 (0.021, 0.064)	0.008 (0, 0.026)	0.046 (0.032, 0.06)	0.011 (0, 0.029)
Sensitivity	*ϕ* (%)	50.9 (47.8, 54.2)	50.2 (46.0, 54.4)	61.8 (59.6, 64.0)	58.6 (56.1, 61.1)
Importation probability	*f* (%)	4.5 (3.1, 6.1)	6.2 (4.8, 7.8)	6.0 (4.8, 7.1)	9.9 (8.2, 11.6)
Fraction colonized	*p*_col_ (%)	24.4 (23.1, 25.8)	19.9 (18.7, 21.2)	22.3 (21.6, 23.0)	24.4 (23.7, 25.1)
**Contributions**					
Background	*R*_background_ (%)	53.6 (32.8, 75.9)	89.3 (67.9, 100)	43.4 (29.1, 58.7)	84.5 (60.9, 100)
Cross-transmission	*R*_crossT_ (%)	46.4 (24.1, 67.2)	10.7 (0, 32.1)	56.6 (41.3, 70.9)	15.5 (0, 39.1)

*Highest posterior density interval

^†^ 20/04/1999 − 23/01/2008 for ICU A and 11/01/2000 − 12/01/2011 ICU B.

^‡^ 31/05/2009 − 30/12/2016 for ICU A and 13/01/2011 − 13/09/2016 for ICU B.

The probability of being colonized with *P. aeruginosa* on admission and the screening test sensitivity varied between the two ICUs and the time periods. For both ICUs, the median estimates of the importation probability *f* is higher in the data set after renovation than before, i.e. 4.5% and 6.2% for ICU A and 6.0% and 9.9% for ICU B. The difference between the time periods is only significant for ICU B. We estimated the median of the prevalence of *P.A.* to be 24.4% and 19.9% for ICU A and 22.3% and 24.4% for ICU B before and after renovation, respectively. Median estimates for the screening test sensitivity were 50.9% and 50.2% for ICU A and 61.8% and 58.6% for ICU B. Since the credibility intervals of the sensitivity estimates do not overlap with respect to the two ICUs, we can conclude that there is a 95% probability that the test sensitivity is higher in ICU B than in ICU A. Our possible explanation is based on the fact that the ICUs differ in their patient population. As a medical ward, ICU B contains patients with longer lengths of stay and more readmissions. Patients who are exposed to an ICU environment for a longer period of time may have a higher probability to get colonized at a detectable level. However, our explanation is only hypothetical and the true reason for the difference is not known.

**Fig 5:**
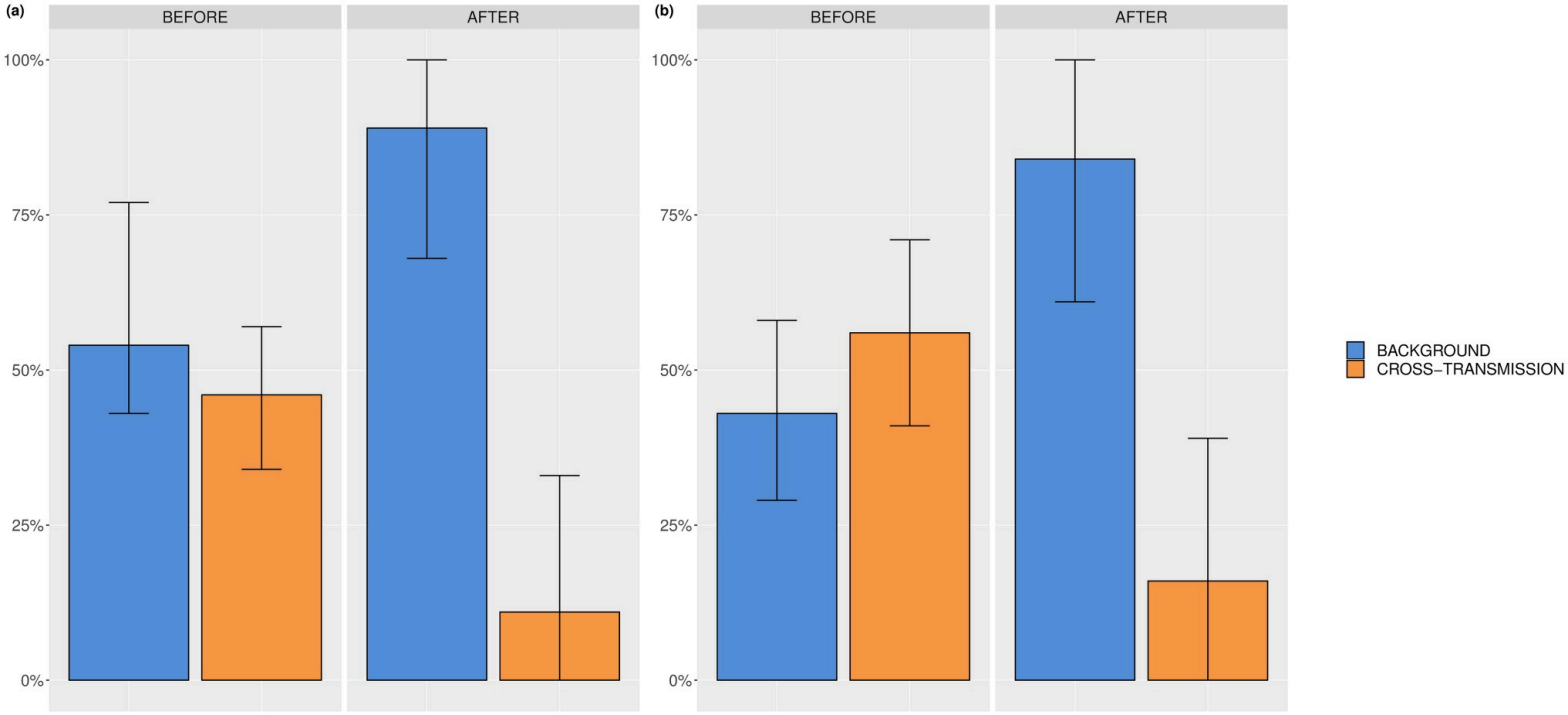
Relative contributions of background and cross-transmission. (a) ICU A before and after renovation, (b) ICU B before and after renovation.

The relative importance of the two considered transmission routes per ICU and time period is depicted in Fig 5(a) and 5(b). For ICU A, the median relative contribution of background transmission is 53.6% (95% CrI: 32.8 − 75.9%) and 89.3% (95% CrI: 67.9 − 100%) leaving 46.4% (95% CrI: 24.1 − 67.2%) and 10.7 (95% CrI: 0 − 32.1%) of the acquisitions assigned to cross-transmission before and after renovation, respectively. For ICU B, 43.4% (95% CrI: 29.1 − 58.7%) and 84.5% (95% CrI: 60.9 − 100%) of the acquisitions were due to the background and cross-transmission accounted for 56.6% (95% CrI: 41.3 − 70.9%) and 15.5% (95% CrI: 0 − 39.1%) of the acquisitions before and after renovation, respectively.

The results suggest that both routes have an important impact on the acquisitions in both ICUs. The median estimates of the relative contribution of background transmission are higher after than before renovation in both ICUs. Thus, there is a tendency for lower contribution of cross-transmission route after renovation in both ICUs. Possibly, hygiene was improved after renovating the ICUs. However, since the credibility interval for background transmission overlap before and after renovation, there is no evidence that the relative contributions differ between the time periods. Before renovation, the credibility intervals of the relative contributions for background and cross-transmission overlap. Thus, we conclude that no route considerably predominates the transmissions before renovation. On the other hand, the respective credibility intervals do not overlap after renovation. Hence, background transmission predominates the transmissions after renovation. Comparing the results across ICUs, we can see that the credibility intervals of the relative contributions overlap leading to the conclusion that the two ICUs do not seem to be different regarding the relative importance of the transmission routes.

#### Full model: Three transmission routes

**Fig 6:**
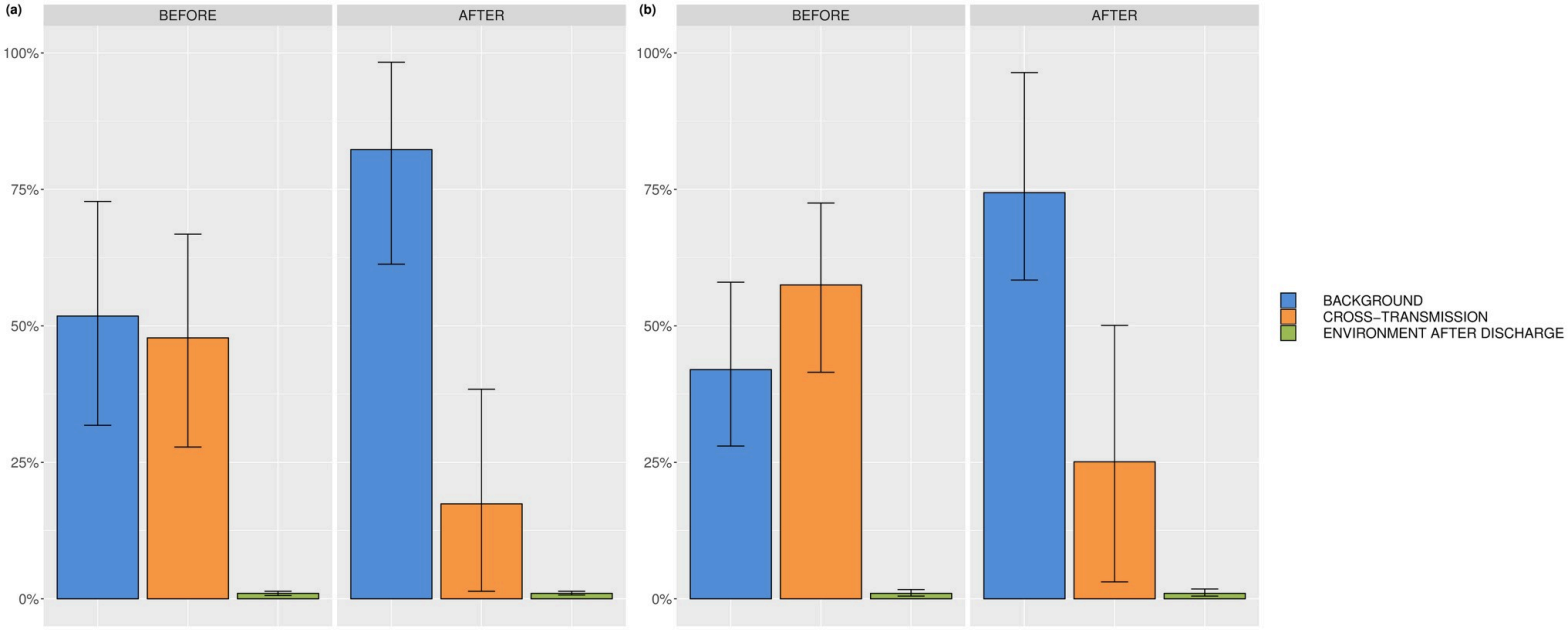
Relative contributions of background transmission, cross-transmission and environmental contamination after discharge. (a) ICU A before and after renovation, (b) ICU B before and after renovation.

Posterior estimates of the model parameters for each ICU are reported in Table 3. The estimates and interpretations for the importation rate *f*, the screening test sensitivity *ϕ* and the mean prevalence stay roughly the same when adding environmental contamination as an additional route. The same holds for the median relative contributions of background and cross-transmission. The median relative contribution of environmental contamination after discharge is less than 1% ranging from 0.3% to 0.5% for both ICUs and both time periods. The relative importance of the three considered transmission routes per ICU and time period is depicted in Fig 6(a) and 6(b). Acceptance probabilities for proposed updates to the augmented data ranged from 7.2% (ICU B after renovation) to 90% (ICU A before renovation). Pairwise scatter plots indicated strong correlations between *α* and *β*, *β* and *ϵ* and between *ϵ* and *μ* (see S14 Fig). The correlation coefficient of the latter pair ranged from 0.531 to 0.561. Furthermore, it can be seen in Table 3 that the credibility intervals for the parameters *ϵ* and *μ* are large. Nevertheless, histogram and trace plots of the posterior estimates show that the MCMC chains rapidly mixed and quickly converged to their stationary distribution as can be seen in S6–S13 Figs. The rapid convergence could be achieved by tuning the parameters of the proposal distribution for *μ*. In contrast, a flat prior for the decay rate *μ* in combination with a small initial standard deviation for its proposal distribution resulted in large acceptance ratios close to 1. The MCMC chain mixed too slowly and therefore hindered the identifiability of the likelihood. This can be explained by the fact that our developed model is overparametrized when colonizations of patients are not or hardly influenced by environmental contamination. Small values of the transmission parameter *ϵ* as well as high values of the decay rate *μ* would reflect the aforementioned situation. As a result, the respective likelihood might not be or only weakly identifiable. Our sensitivity analyses and artificial data simulations demonstrated similar pairwise scatter plots and wide credibility intervals for the parameters *ϵ* and *μ* in case of a small contribution of environmental contamination to the transmissions (more details can be found in S9 Text). Hence, we can conclude that the role of environmental contamination after discharge within the transmission process of P. aeruginosa in the two ICUs A and B is small before as well as after renovation.

**Table 3:** Summary statistics of the marginal posterior distributions for parameters of the full model based on the analysis of the Besançon data.

Parameter	Symbol	Median (95% credibility interval)*			
		ICU A		ICU B	
		Before ^†^	After ^‡^	Before	After
Background coefficient	*α*	0.011 (0.006, 0.015)	0.012 (0.009, 0.016)	0.007 (0.004, 0.01)	0.012 (0.007, 0.017)
Cross-transmission coefficient	*β*	0.023 (0, 0.048)	0.006 (0, 0.022)	0.027 (0.0, 0.05)	0.008 (0, 0.027)
Environmental coefficient	*ϵ*	187.3 (0.041, 753.4)	76.0 (0002, 436.6)	48.8 (0.009, 227.9)	90.6 (0.017, 502.4)
Decay rate	*μ*	1202.7 (24.4, 3184.9)	1567.3 (34.9, 4453.7)	319.2 (13.8, 873.3)	1514.2 (44.4, 4335.1)
Sensitivity	*ϕ* (%)	50.9 (47.8, 54.2)	49.5 (45.5, 53.5)	61.8 (59.5, 64.0)	58.7 (56.0, 61.3)
Importation probability	*f* (%)	4.5 (3.1, 6.1)	6.1 (4.9, 7.6)	6.0 (4.9, 7.2)	10.1 (8.4, 11.9)
Fraction colonized	*p*_col_ (%)	24.5 (23.1, 25.9)	20.1 (19.0, 21.4)	22.3 (21.7, 23.0)	24.4 (23.7, 25.1)
**Contributions**					
Background	*R*_background_ (%)	51.8 (32.7, 73.0)	82.3 (61.0, 98.7)	42.0 (27.5, 58.0)	74.4 (48.1, 96.8)
Cross-transmission	*R*_crossT_ (%)	47.8 (26.9, 66.9)	17.4 (1.3, 38.6)	57.5 (41.8, 72.1)	25.1 (2.9, 50.7)
Env. cont. after discharge	*R*_env_ (%)	0.3 (0.0, 0.8)	0.2 (0, 0.7)	0.5 (0.0, 1.2)	0.4 (0.0, 1.3)

*Highest posterior density interval

^†^ 20/04/1999 − 23/01/2008 for ICU A and 11/01/2000 − 12/01/2011 ICU B.

^‡^ 31/05/2009 − 30/12/2016 for ICU A and 13/01/2011 − 13/09/2016 for ICU B.

### Model selection

In total, 14 analyses were performed. For each ICU, three data sets were created—one for each time period and one combining the data sets before and after renovation. Additionally, the ICUs and time periods were combined in one data set. Each of the seven data sets were analyzed using the submodel and the full model. The DIC values for each model analysis can be found in Table 4. The analysis combining both ICUs and time periods shows smaller DIC values, i.e. 136507.8 and 130693, than the sum of the DICs for separate analyses (152428.8 and 150914.2) for both the submodel and full model, respectively. The full model results in a smaller DIC value for the analysis of the combined data set. Hence, based on the DIC, it would be sufficient to analyze the combined data set using the full model including endogenous route, cross-transmission and environmental contamination. Nevertheless, it can be seen in S10 Text that the posterior estimates of the different analyses are similar, especially for the relative contribution of environmental contamination after discharge.

**Table 4:** Deviance information criterion for the different models*.

	Submodel		Full model	
**ICU A**				
Before ^†^	42961.72	∑^¶^ 86899.53	35356.31	∑ 63057.37
After ^‡^	43937.81		27701.06	
Combined ^§^	88650.69		63779.12	
**ICU B**				
Before	35356.31	∑ 63057.37	34670.46	∑ 62568.88
After	27701.06		27898.42	
Combined	63778.12		59290.73	
**Sum** Combined ^‖^	152428.8		150914.2	
**ICUs combined** ^#^	136507.8		130693	

* Computation based on S6 Text.

^†^ 20/04/1999 − 23/01/2008 for ICU A and 11/01/2000 − 12/01/2011 ICU B.

^‡^ 31/05/2009 − 30/12/2016 for ICU A and 13/01/2011 − 13/09/2016 for ICU B.

^§^ Combined time periods.

^¶^ Σ indicates that the sum of the respective rows in the previous column is calculated.

^‖^ The sum of DICs for ICU A (before and after combined) and ICU B is computed.

^#^ ICUs as well as time period (before and after) are combined in one data set.

### Model assessment

**Fig 7:**
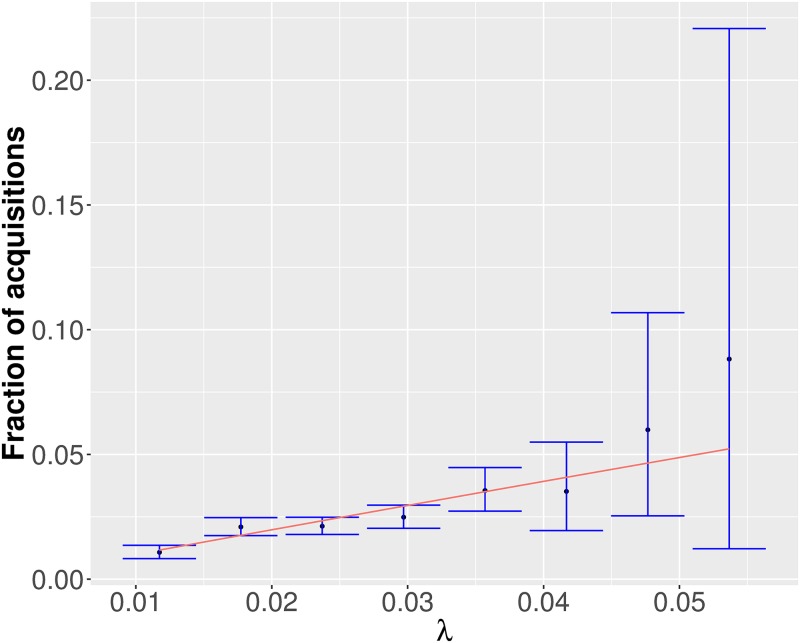
Exemplary model assessment plot for one MCMC update using the submodel applied to ICU A before renovation. The predicted fraction of acquisition is plotted against the theoretical force of infection. The red line indicates the theoretical relation between the force of infection and the probability of acquisition. The blue lines indicate 95% credibility intervals.

For each bin of the force of infection the coverage probabilities are plotted and can be found in S15 and S16 Figs. It can be seen that the coverage probabilities are approximately (sometimes higher, sometimes smaller) equal to the nominal confidence level of 0.95. Thus, both the full model as well as the submodel gave adequate fits to the four data sets. In Fig 7, the predicted fraction of acquisitions are plotted against the binned force of infection for one exemplary MCMC update. The red lines indicate the relationship between the probability of acquisition and force of infection assumed by our models. For this example, it is always contained by the confidence intervals (blue lines).

## Discussion

To our knowledge, our study is the first attempt to estimate the relative contribution of environmental contamination after discharge for *P. aeruginosa* based on mathematical modeling and using only admission, discharge and screening data. The three different routes, background transmission, cross-transmission and environmental contamination after discharge, are distinguished by the resulting patterns of the prevalence that they induce. We estimated that environmental contamination after discharge accounts for at most 1% of the total *P. aeruginosa* transmissions in the two ICUs of the University hospital in Besançon before and after renovation. In contrast, background as well as cross-transmission are both essential for the transmission dynamics of *P. aeruginosa*. This suggests, that improvement of cleaning of the environment *after discharge* would have only a limited benefit regarding the prevention of *P. aeruginosa* colonization in the two considered ICUs of the University hospital in Besançon.

Previously, studies have been conducted to investigate the role of environmental contamination for colonizations of *P. aeruginosa*. For instance, Panagea et al. performed environmental studies to determine the extent of environmental contamination with an epidemic strain of *P. aeruginosa* [35]. They concluded that the transmissibility of the epidemic strain cannot be explained solely on the basis of improved environmental survival. Our results likewise demonstrate that the decay of *P. aeruginosa* is already rapid enough to limit its survival in the environment.

While our approach is efficient in determining the relative contribution of environmental contamination after discharge requiring merely longitudinal surveillance data, it has several limitations that may restrict its practical applicability.

Our conclusions on the impact of cleaning applies only to the environment after the discharge of patients. Permanently contaminated reservoirs in ICUs, such as sinks, may still serve as sources for colonization. In our model they are assigned to background transmission. Thus, while the effect of cleaning improvement after discharge might be limited for the two considered ICUs, general cleaning improvement of the environment might be important to reduce permanent reservoirs for environmental contamination. Several studies based on molecular typing techniques suggest that contaminated taps and sinks in the environment may serve as a non-negligible source in the acquisition of *P. aeruginosa* colonization (see e. g. [8, 21, 36]). Since genotyping information is not available for the data set that we have analyzed, further studies for validation would increase the understanding of our conclusions.

In addition, we assume in our model that a patient’s contribution to environmental contamination affects *all* patients present in the ward. This assumption might not be realistic as the patient admitted to the same room after the discharge of a colonized patient might be at a higher risk than patients in other rooms. In S11 Text, we have investigated the possible influence of prior colonized bed occupants for the Besançon data sets. The results show that for these data sets, the impact of prior colonized bed occupants is limited (< 6%). While prior bed occupants may pose serious risks for colonization in general, this hypothesis cannot be confirmed for the data sets we have analyzed. Further models that explore bedwise environmental contamination in more detail would constitute interesting extensions of our methodology.

The results of our analysis build on a data-augmented MCMC algorithm [27, 30]. Markov chain Monte Carlo sampling is a powerful tool to estimate posterior parameter distribution whenever the likelihood is analytically intractable. And yet, the inherent disadvantage of this sampling scheme is that it may take prohibitively many iterations to converge to the posterior distribution. The convergence properties of MCMC sampling in high-dimensional posterior distributions can be particularly problematic and sensitive to the choice of prior and proposal distributions. Thus, tuning of the MCMC parameters becomes crucial for its application. Our developed full model is overparametrized when colonizations of patients are not or hardly influenced by environmental contamination. As a result, the respective likelihood might not be identifiable or only weakly identifiable. Here, a flat prior for the decay rate *μ* in combination with a small initial standard deviation for its proposal distribution resulted in large acceptance ratios close to 1. The MCMC chain mixed too slowly and therefore hindered the identifiability of the likelihood. We were able to tune the parameters of the proposal distribution for *μ* such that rapid convergence to the posterior distribution could be assessed using visual inspection of histograms and trace plots. However, as presented in the *Results* section, pairwise scatter plots showed strong correlations in particular between *ϵ* and *μ*. Simulation studies confirmed that this can be explained by an absence of environmental contamination in the investigated data sets. This supports our finding that an impact of environmental contamination after discharge on the transmission of *P. aeruginosa* may be neglected.

Moreover, colonization is assumed to remain until discharge. While this assumption is true for *P. aeurginosa* it does not hold true for all antibiotic-resistant nosocomial pathogens. However, intermittent carriage may be readily included allowing the method to be generalized to other pathogens.

We assumed no difference in transmissibility between different strains of *P. aeruginosa* and that all colonized patients are equally likely to transmit the pathogen. While information on antibiotic resistance or microbial genotyping in combination with epidemiological data may aid in distinguishing different strains and identifying specific transmission events, only the uncertainty of the estimates would be affected. In particular, the widths of the credibility intervals are likely to be reduced, but we do not expect a large effect on the parameter estimates.

Assessing the fit of the model to the data is crucial to model building. The true relative importance of the different transmission routes in ICUs is generally unknown. Genotyping data that might be used to demonstrate the source of the acquired colonization is scarce and was not available for the data used in our analysis. While the posterior predictive *p*-value is a popular method for assessing model fit, it has been increasingly criticized for its self-fulfilling nature [37]. Furthermore, the choice of the test statistic is crucial in order to adequately summarize discrepancies between datasets. Rather than relying on a suitable summary statistic, we presented a model assessment method that evaluates whether the estimated force of infection adequately represents the transmission dynamics in the ward. However, while the corresponding coverage probabilities may depict discrepancies per bin of the force of infection, the sample size is not controlled by choosing the number MCMC updates. It might well occur that specific patients (and their acquisition events) appear in more than one MCMC update simultaneously. Thus, the true sample size is estimated to be smaller. In addition, both the estimated force of infection and the number of acquisitions *N*_acq_ are obtained based on the data augmentation step. Thus, the theoretical probability of acquisition and the predicted fraction of acquisition are not independent. And yet, a large deviation of the model from the data would be reflected in the coverage probabilities since the augmented data is dependent on the observed data. Further improvement of the method presented here or development of other methods would be a vital topic for assessing epidemic models.

Model selection was performed using the DIC which is known to display poor performance (i.e. identifying the correct model) for complex likelihood functions such as those corresponding to epidemic models. Comparing the plausibility of different models is crucial for selecting the model that describes the dynamics of the observed system best. Nevertheless, model choice for stochastic epidemic models is far from trivial. All known approaches for model selection exhibit advantages as well as disadvantages [37] which makes selecting the most suitable model comparison technique not straightforward. We selected the well-known DIC-method that was easy to use and implement. Our main results regarding environmental contamination after discharge do not depend on the model choice. And yet, the development of a suitable and robust model selection procedure in a data-augmented Bayesian framework would be an interesting and important topic for future research.

Finally, like all models, ours is a simplification of the truth as it is unlikely that all relevant variables are already included. Adding covariates such as antibiotic use, sex or age may improve the model fit.

Our work may be used or further extended for assessing the relative importance of different transmission routes within intensive-care units not only for *P. aeruginosa* but for hospital pathogens in general. Based on these results, consequential decisions for tailored interventions or policies may be deduced, aiding in improving infection prevention and control and therefore reducing morbidity, mortality and related costs in hospitals.

## Supporting information

S1 Text Environmental contamination. (PDF)

S2 Text Discrete-time transmission model. (PDF)

S3 Text Relative contribution. (PDF)

S4 Text Approximation of relative contribution in discrete-time. (PDF)

S5 Text Adapted data-augmented MCMC algorithm. (PDF)

S6 Text Model selection. (PDF)

S7 Text Model assessment. (PDF)

S8 Text Prior distributions. (PDF)

S9 Text Simulation studies. (PDF)

S10 Text Secondary analyses. (PDF)

S11 Text Impact of prior colonized bed occupants. (PDF)

S12 Text COMBACTE-MAGNET membership list. (PDF)

S1 Table Summary statistics of the marginal posterior distributions for parameters of the submodel based on the analysis of the Besançon data. (PDF)

S2 Table Summary statistics of the marginal posterior distributions for parameters of the full model based on the analysis the Besançon data. (PDF)

S3 Table Association of colonization statuses of consecutive bed occupants in ICU A of the University hospital of Besançon. (PDF)

S4 Table Association of colonization statuses of consecutive bed occupants in ICU B of the University hospital of Besançon. (PDF)

S5 Table Summary statistics of the marginal posterior distributions for parameters of model (7) based on the analysis of the Besançon data. (PDF)

S1 Fig Pairwise plots of samples from the posterior distribution for the transmission parameters of the submodel. The plots were generated from the data of ICU A before renovation using the submodel with background and cross-transmission.
(TIF)

S2 Fig Histograms for ICU A before renovation using the submodel with background and cross-transmission. (TIF)

S3 Fig Traceplots for ICU A before renovation using the submodel with background and cross-transmission. (TIF)

S4 Fig Histograms for ICU A after renovation using the submodel with background and cross-transmission. (TIF)

S5 Fig Traceplots for ICU A after renovation using the submodel with background and cross-transmission. (TIF)

S6 Fig Histograms for ICU A before renovation using the full model with background, cross-transmission and environmental contamination. The results are displayed for transmission parameters *α*, *β*, *ϵ* and *μ*.
(TIF)

S7 Fig Histograms for ICU A before renovation using the full model with background, cross-transmission and environmental contamination. The results are displayed for the importation probability *f*, sensitivity parameter *ϕ*, relative contributions *R*_*i*_, *i* ∈ {background, cross- transmission, environment} and log-likelihood.
(TIF)

S8 Fig Traceplots for ICU A before renovation using the full model with background, cross-transmission and environmental contamination. The results are displayed for transmission parameters *α*, *β*, *ϵ* and *μ*.
(TIF)

S9 Fig Traceplots for ICU A before renovation using the full model with background, cross-transmission and environmental contamination. The results are displayed for the importation probability *f*, sensitivity parameter *ϕ*, relative contributions *R*_*i*_, *i* ∈ {background, cross- transmission, environment} and log-likelihood.
(TIF)

S10 Fig Histograms for ICU A after renovation using the full model with background, cross-transmission and environmental contamination. The results are displayed for transmission parameters *α*, *β*, *ϵ* and *μ*.
(TIF)

S11 Fig Histograms for ICU A after renovation using the full model with background, cross-transmission and environmental contamination. The results are displayed for the importation probability *f*, sensitivity parameter *ϕ*, relative contributions *R*_*i*_, *i* ∈ {background, cross- transmission, environment} and log-likelihood.
(TIF)

S12 Fig Traceplots for ICU A after renovation using the full model with background, cross-transmission and environmental contamination. The results are displayed for transmission parameters *α*, *β*, *ϵ* and *μ*.
(TIF)

S13 Fig Traceplots for ICU A after renovation using the full model with background, cross-transmission and environmental contamination. The results are displayed for the importation probability *f*, sensitivity parameter *ϕ*, relative contributions *R*_*i*_, *i* ∈ {background, cross- transmission, environment} and log-likelihood.
(TIF)

S14 Fig Pairwise plots of samples from the posterior distribution for the transmission parameters of the full model. The plots were generated from the data of ICU A before renovation using Exp(0.001) prior and the full model with background, cross-transmission and environmental contamination.
(TIF)

S15 Fig Coverage probabilities for the submodel using Jeffreys prior. (a)—(b) ICU A before and after renovation, respectively. (c)—(d) ICU B before and after renovation, respectively.
(TIF)

S16 Fig Coverage probabilities for the full model using Jeffreys prior. (a)—(b) ICU A before and after renovation, respectively. (c)—(d) ICU B before and after renovation, respectively.
(TIF)

S17 Fig Pairwise plots of samples from the posterior distribution for the transmission parameters of the full model. The plots were generated from the data of ICU A using U(0,2) prior and the full model with background, cross-transmission and environmental contamination after discharge.
(TIF)

S18 Fig Coverage probabilities for simulated data set using Jeffreys prior. The data was simulated using *α* = 0.015, *β* = 0.055, *μ* = 1/7, *ϵ* = 0.15, *f* = 0.05, *ϕ* = 1. The analysis assumed only one route, i.e. background transmission. The plot shows large discrepancies between the expected and the computed coverage probabilities, pointing to a misspecified model.
(TIF)
