# Supplementary material for: Tracking Pseudomonas aeruginosa transmissions due to environmental contamination after discharge in ICUs using mathematical models
Source: PLoS Comput Biol. 2019 Aug 28;15(8):e1006697. doi: 10.1371/journal.pcbi.1006697 (PMC6736315; doi:10.1371/journal.pcbi.1006697)
Supplement: S1 Text — (PDF) [file pcbi.1006697.s001.pdf]

Supporting information

S1 Text. Environmental contamination. The full model includes environmental contamination on a ward-level. The bacterial load at any given time t is based on the differential equation

$$\frac{dE}{dt} = \nu \frac{I(t)}{N(t)} - \mu E(t) \tag{1}$$

Solving the above differential equation requires discretizing over t, resulting in a finite number of time steps $t_0, t_1, \ldots, t_N$. We then assume $I(t) = I_t$ and $N(t) = N_t$ to be constant within a time step and use it as initial conditions. Separating variables leads to

$$\frac{dE}{\nu \cdot \frac{I_t}{N_t} - \mu E(t)} = dt$$

and thus

$$\begin{aligned}
&\int \frac{dE}{\nu \frac{I_t}{N_t} - \mu E(t)} = \int dt \\
\Rightarrow &-\frac{1}{\mu} \log \left| \nu \frac{I_t}{N_t} - \mu E(t) \right| = t + C \\
\Rightarrow &\log \left| \nu \frac{I_t}{N_t} - \mu E(t) \right| = -\mu(t + C) \\
\Rightarrow &\left| \nu \frac{I_t}{N_t} - \mu E(t) \right| = \exp[-\mu(t + C)] = \exp(-\mu t) \underbrace{\exp(-\mu C)}_{A_t}
\end{aligned}$$

Now, two cases have to be distinguished.

1. Case: $\nu \frac{I_t}{N_t} - \mu E(t) \geq 0$

$$\begin{aligned}
\Rightarrow &-\mu E(t) = A_t \cdot \exp[-\mu t] - \nu \frac{I_t}{N_t} \\
\Rightarrow &E(t) = \underbrace{-\frac{A_t}{\mu}}_{B_t} \exp[-\mu t] + \frac{\nu}{\mu} \frac{I_t}{N_t} \\
\Rightarrow &E(t) = B_t \cdot \exp[-\mu t] + \frac{\nu}{\mu} \frac{I_t}{N_t}.
\end{aligned}$$

2. Case: $\nu \frac{I_t}{N_t} - \mu E(t) < 0$

$$\Rightarrow \mu E(t) = A_t \cdot \exp[-\mu t] + \nu \frac{I_t}{N_t}$$

$$\Rightarrow E(t) = \underbrace{\frac{A_t}{\mu}}_{B_t} \exp[-\mu t] + \frac{\nu}{\mu}\frac{I_t}{N_t}$$

$$\Rightarrow E(t) = B_t \cdot \exp[-\mu t] + \frac{\nu}{\mu}\frac{I_t}{N_t}.$$

Determine B_{t_0} for initial condition $E(t_0) = E_{t_0}$:

$$E_{t_0} = E(t_0) = B_0 + \frac{\nu}{\mu}\frac{I_{t_0}}{N_{t_0}}$$

and therefore

$$B_{t_0} = E_{t_0} - \frac{\nu}{\mu}\frac{I_{t_0}}{N_{t_0}}. \tag{2}$$

For $t_0 \leq t \leq t_1$ the environmental load can be then computed by

$$\begin{aligned} E(t) &= \left(E_{t_0} - \frac{\nu}{\mu}\frac{I_{t_0}}{N_{t_0}}\right) e^{-\mu t} + \frac{\nu}{\mu}\frac{I_{t_0}}{N_{t_0}} \\ &= E_{t_0} e^{-\mu t} + \frac{\nu}{\mu}\frac{I_{t_0}}{N_{t_0}}(1 - e^{-\mu t}). \end{aligned}$$

For $t_0 \leq t_i \leq t_N$ it holds

$$B_{t_i} = \frac{E_{t_i} - \frac{\nu}{\mu}\frac{I_{t_i}}{N_{t_i}}}{e^{-\mu t_i}} \tag{3}$$

and therefore, it holds for $\lfloor t \rfloor := \max\{t_0 \leq x \leq t_N \,|\, x \leq t\}$ and $t \in \mathbb{R} \setminus \{t_0, t_1, \ldots, t_N\}$

$$\begin{aligned} E(t) &= \frac{E_{\lfloor t \rfloor} - \frac{\nu}{\mu}\frac{I_{\lfloor t \rfloor}}{N_{\lfloor t \rfloor}}}{e^{-\mu \lfloor t \rfloor}} \cdot e^{-\mu t} + \frac{\nu}{\mu}\frac{I_{\lfloor t \rfloor}}{N_{\lfloor t \rfloor}} \\ &= E_{\lfloor t \rfloor} e^{-\mu(t - \lfloor t \rfloor)} + \frac{\nu}{\mu}\frac{I_{\lfloor t \rfloor}}{N_{\lfloor t \rfloor}}\left(1 - e^{-\mu(t - \lfloor t \rfloor)}\right). \end{aligned}$$

and

$$E(t_i) = E_{t_{i-1}} e^{-\mu(t_i - t_{i-1})} + \frac{\nu}{\mu}\frac{I_{t_{i-1}}}{N_{t_{i-1}}}\left(1 - e^{-\mu(t_i - t_{i-1})}\right)$$

for $0 \leq i \leq N$.
