# Supplementary material for: Tracking Pseudomonas aeruginosa transmissions due to environmental contamination after discharge in ICUs using mathematical models
Source: PLoS Comput Biol. 2019 Aug 28;15(8):e1006697. doi: 10.1371/journal.pcbi.1006697 (PMC6736315; doi:10.1371/journal.pcbi.1006697)
Supplement: S2 Text — (PDF) [file pcbi.1006697.s002.pdf]

S2 Text. Discrete-time transmission model. For the discrete-time transmission model, we assume that the number of colonized patients $I(t)$, the total number of patients $N(t)$ and the bacterial load $E(t)$ is constant during the day. It is assumed that admission and screening occur at 12:00 pm on each day T determining I_T and N_T. Given all the information (at 12:00 pm), the environmental contamination on day T is determined. The force of infection on day T is then given by

$$\begin{aligned}\lambda(T) &= \alpha + \beta\frac{I_T}{N_T} + \epsilon E(T) \\ &= \alpha + \beta\frac{I_T}{N_T} + \epsilon \cdot \left[E_{T-1}e^{-\mu} + \frac{\nu}{\mu}\frac{I_{T-1}}{N_{T-1}}(1-e^{-\mu})\right].\end{aligned}$$
