# Supplementary material for: Tracking Pseudomonas aeruginosa transmissions due to environmental contamination after discharge in ICUs using mathematical models
Source: PLoS Comput Biol. 2019 Aug 28;15(8):e1006697. doi: 10.1371/journal.pcbi.1006697 (PMC6736315; doi:10.1371/journal.pcbi.1006697)
Supplement: S3 Text — (PDF) [file pcbi.1006697.s003.pdf]

S3 Text. Relative contribution. The computations in section *Relative contributions of transmission routes* were developed for continuous-time models. In our discrete-time model, we assume that events such as, admission, colonization and discharge of patients and screening occur on a daily basis. However, we do assume that the level of environmental contamination changes continuously. Computing the relative contributions of the different transmission routes becomes more laborious in this scenario. Let t_i^c be the acquisition time of patient $i \in \{1, \ldots, n\}$. The contribution of a route j is the ratio of the probability that the acquisition was due to route j and the total probability of acquisition:

$$\text{Contribution of route } j = R_j = \frac{\sum_{i=1}^{n} \frac{P(\text{infection during day } t_i^c \text{ due to route } j)}{P(\text{infection during day } t_i^c)}}{N_{\text{acq}}}$$

where N_{acq} is the total number of occured colonizations and R_j with $j \in \{\text{background}, \text{crossT}, \text{env}\}$ indicate the endogenous, cross-transmission or environmental route, respectively. The route-specific probabilities can be determined by

$$P(\text{infection during day } T \text{ by } R_{\text{background}}) = \int_T^{T+1} \text{P(patient still susceptible at time } t) \cdot \alpha \, dt$$
$$P(\text{infection during day } T \text{ by } R_{\text{crossT}}) = \int_T^{T+1} \text{P(patient still susceptible at time } t) \cdot \beta \frac{I_T}{N_T} \, dt$$
$$P(\text{infection during day } T \text{ by } R_{\text{env}}) = \int_T^{T+1} \text{P(patient still susceptible at time } t) \cdot \epsilon E(t) \, dt$$

where environmental contamination during a patient's stay is assigned to the environmental route. In the main part of our manuscript we consider only the bacterial load remaining after discharge as environmental contamination. All the formulas then change according to Eq. (3) of the main text.
The results are dependent on $\nu \frac{I_T}{N_T} - \mu E(T)$ and are given by

1. Case: $\nu \frac{I_T}{N_T} - \mu E(T) \geq 0$

$$P(\text{infection during day } T \text{ by } R_{\text{background}}) = \alpha \cdot \tilde{E} \cdot \left[\gamma\left(\frac{A}{\mu}, \tilde{B}\right) - \gamma\left(\frac{A}{\mu}, \tilde{B} e^{-\mu}\right) \right]$$
$$P(\text{infection during day } T \text{ by } R_{\text{crossT}}) = {}_{\text{,}}\beta \frac{I_T}{N_T} \cdot \tilde{E} \cdot \left[\gamma\left(\frac{A}{\mu}, \tilde{B}\right) - \gamma\left(\frac{A}{\mu}, \tilde{B} e^{-\mu}\right) \right]$$
$$P(\text{infection during day } T) = 1 - e^{-A - \tilde{B}\left(e^{-\mu} - 1\right)}$$

2. Case: $\nu \frac{I_T}{N_T} - \mu E(T) < 0$

$$P(\text{infection during day } T \text{ by } R_{\text{background}}) = \alpha \cdot \hat{E} \cdot \left[\sum_{i=0}^{\infty} \frac{(-\tilde{B})^i}{i!} \cdot \frac{1}{A/\mu + i} \cdot \left(1 - e^{-A-\mu i}\right) \right]$$

$$P(\text{infection during day } T \text{ by } R_{\text{crossT}}) = \text{¸}\beta \frac{I_T}{N_T} \cdot \hat{E} \cdot \left[\sum_{i=0}^{\infty} \frac{(-\tilde{B})^i}{i!} \cdot \frac{1}{A/\mu + i} \cdot \left(1 - e^{-A-\mu i}\right) \right]$$

$$P(\text{infection during day } T) = 1 - e^{-A-\tilde{B}\left(e^{-\mu}-1\right)}$$

and

$$P(\text{infection during day } T \text{ by } R_{\text{background}})$$
$$= P(\text{infection during day } T) - \sum_{k \in \{\alpha,\beta\}} P(\text{infection during day } T \text{ by } k)$$

where

$$A = \alpha + \left(\beta + \frac{\epsilon\nu}{\mu}\right) \frac{I_T}{N_T}, \qquad \tilde{B} = \frac{\epsilon}{\mu}\left(\frac{\nu}{\mu}\frac{I_T}{N_T} - E_T\right)$$

$$\tilde{E} = \frac{1}{\mu} \cdot e^{\tilde{B}} \cdot \tilde{B}^{-\frac{A}{\mu}}, \qquad \hat{E} = \frac{e^{\tilde{B}}}{\mu},$$

$$G = \frac{\epsilon\nu}{\mu}\frac{I_T}{N_T}$$

and $\gamma(\cdot,\cdot)$ is the lower incomplete gamma function. Note that the derivations are omitted here but can be requested from the first author.
