# Supplementary material for: Tracking Pseudomonas aeruginosa transmissions due to environmental contamination after discharge in ICUs using mathematical models
Source: PLoS Comput Biol. 2019 Aug 28;15(8):e1006697. doi: 10.1371/journal.pcbi.1006697 (PMC6736315; doi:10.1371/journal.pcbi.1006697)
Supplement: S4 Text — (PDF) [file pcbi.1006697.s004.pdf]

S4 Text. Approximation of relative contribution in discrete-time. Large values of the force of infection $\lambda(t)$ are very unlikely. Under the assumption of small $\lambda(t)$, the following simplifications and approximations can be made:

$$
\begin{aligned}
e^{-\int_0^t \lambda(x)dx} &\approx 1 - \lambda(t) \\
1 - e^{-\int_0^t \lambda(x)dx} &\approx \lambda(t) \\
\frac{\lambda(t)}{1 - \lambda(t)} &\approx \lambda(t).
\end{aligned}
$$

Therefore, the force of infection itself may be a good approximation of the probability of infection and the probability of acquiring colonization due to route j may be approximated by the respective sub-term of the force of infection assigned to route j:

$$P(\text{infection during day } T \text{ due to route } j) \approx \lambda_j$$

with $j \in \{\text{background}, \text{crossT}, \text{env}\}$. As an approximation of the relative contribution we compute the ratio of the transmission rate and the force of infection for each acquired colonization:

- Contribution of endogenous route $= R_{\text{background}} = \frac{\sum_{i=1}^{n} \frac{\alpha}{\lambda(t_i^c)}}{N_{\text{acq}}}$
- Contribution of cross-transmission $= R_{\text{crossT}} = \frac{\sum_{i=1}^{n} \frac{\beta \cdot \frac{I(t_i^c)}{N(t_i^c)} + \epsilon \sum_{i_p} \frac{E_{i_p}(t_i^c)}{N(t_i^c)}}{\lambda(t_i^c)}}{N_{\text{acq}}}$
- Contribution of environmental contamination $= R_{\text{env}} = \frac{\sum_{i=1}^{n} \frac{\epsilon \left[\sum_{i_d} \frac{E_{i_d}(t_i^c)}{N(t_i^c)} + E_0 e^{-\mu t_i^c}\right]}{\lambda(t_i^c)}}{N_{\text{acq}}}$

where t_i^c is the day of colonization of patient $i \in \{1, \dots, n\}$ and N_{acq} the total number of occured colonizations. Furthermore, i_p indicates a colonized patient that is present at time t_i^c and i_d a colonized patient that has been colonized prior to t_i^c but was already discharged. It holds $R_{\text{background}} + R_{\text{crossT}} + R_{\text{env}} = 1$.
