# Supplementary material for: Tracking Pseudomonas aeruginosa transmissions due to environmental contamination after discharge in ICUs using mathematical models
Source: PLoS Comput Biol. 2019 Aug 28;15(8):e1006697. doi: 10.1371/journal.pcbi.1006697 (PMC6736315; doi:10.1371/journal.pcbi.1006697)
Supplement: S5 Text — (PDF) [file pcbi.1006697.s005.pdf]

S5 Text. Adapted data-augmented MCMC algorithm. We model the transmission process using a two-state Markov model, where each patient can be either *susceptible* ($P. A.$ negative) or *colonized* ($P. A.$ positive). A patient i is admitted to the ICU on day t_i^a and discharged on day t_i^d. The probability that a patient is admitted already colonized is described by parameter f. The rate at which a susceptible patient transitions to being colonized is described in section *Materials and Methods*. The colonization state of an individual patient is determined from screening information. We suppose that for each patient i a set of screening results $X_i = X_i^{(1)}, \ldots, X_i = X_i^{(m)}$, taken on days $t_i^{(1)}, \ldots, t_i^{(m)}$ is available. The set of all screening results is denoted by $X = \{X_1, \ldots, X_N\}$ where N is the total number of patients. Since screening tests are typically intermittent and imperfect, we define the test sensitivity ϕ (i.e. probability that a colonized patient has a positive result). We assume that the specificity (i.e. probability that an uncolonized patient has a negative result) is 100%.

We implemented an adapted version of the data-augmented MCMC algorithm to analyze the data. The transmission and importation model, as well as the data-augmentation method is closely based on the approach of [27, 30] but adapted for the transmission routes presented in this paper. The algorithm was implemented in C++ and the analysis of the output was performed in `R` (Version 3.5.1) [31]. The aim of our analysis was to estimate the set of parameters $\theta = \{\alpha, \beta, \epsilon, \mu, f, \phi\}$. The prior distribution were chosen as follows:

$$f, \phi \propto \text{Beta}(a, b)$$
$$\alpha, \beta, \epsilon, \mu \propto \text{Exp}(\lambda)$$

where $\text{Exp}(\lambda)$ represents the exponential distribution with rate λ, and $\text{Beta}(a, b)$ the beta distribution with shape parameters a and b. Having fixed $a = b = 1$ and $\lambda = 0.001$, we use uninformative priors in our analysis.

The data-augmentation procedure accounts for unobserved colonization times by augmenting the parameter space with $A = \{t^c, s^a\}$, a set comprising of the unobserved colonization times t^c and admission states s_a of all n patients. An admission state of a patient is 1 if the patient is colonized upon admission and 0 otherwise. If the patient j becomes colonized during his/her stay, the colonization time may take an integer value between the time of admission t_j^a and time of discharge t_j^d (inclusive). If a patient does not acquire colonization, the respective value t_j^c takes a dummy value of -1. The augmented posterior density relation can be determined using Bayes' Theorem:

$$P(A, \theta \mid D) \propto P(D, A, \theta) = P(D \mid A, \theta) P(A \mid \theta) P(\theta) \tag{4}$$
$$= P(D \mid t^c, s^a, \theta) P(s^a \mid \theta) P(t^c \mid s^a, \theta) P(\theta) \tag{5}$$

where $P(D \mid A, \theta)$ is the likelihood of the observed data D, $P(A \mid \theta)$ is the likelihood of the augmented data and

$P(\theta)$ is the joint prior distribution of the parameter set θ. All terms in (4) can be explicitly calculated. It holds

$$P(D \mid t^c, s^a, \theta) = \phi^{TP(X)}(1-\phi)^{FN(X,t^c)}$$

where $TP(X)$ and $FN(X, A)$ are the total number of true positive and false negative swab results, given the colonization times t^c, respectively. It represents the imperfect observation of the transmission dynamics. Assuming that lost colonization can be excluded, we consider any negative result after the time of colonization as a false negative. Since false positive results are impossible, the $TP(X)$ is not dependent on the augmented data and can be determined directly from the observed data. The probability of the set of importations, given the importation probability f is given by

$$P(s^a \mid \theta) = f^{\sum_i s_i^a}(1-f)^{n-\sum_i s_i^a}.$$

The transmission model itself is reflected in the probability of the colonization times given the admission states and the parameters

$$P(t^c \mid s^a, \theta) = \prod_{i=1}^{n} \exp\left(-\sum_{t=t_i^a}^{\min(t_i^d, t_i^c-1)} \lambda(t)\right)\left(\prod_{j:t_j^c \neq -1} (1-\exp(-q(t_j^c)))\right) \cdot f^{\sum_i s_i^a}(1-f)^{n-\sum_i s_i^a}. \quad (6)$$

To update the importation rate f and the sensitivity ϕ, we use Gibbs sampling as we can sample directly from the full conditional distributions. The transmission parameters $\alpha, \beta, \epsilon, \mu$ are updated using an adapted version of the Metropolis-Hastings algorithm. Regular MCMC methods based on the Metropolis-Hastings algorithm tend to be very slow in high dimensions as a result of slow mixing and therefore inefficient convergence towards the target distribution. In high-dimensional spaces the volume outside is much larger than the volume of our target distribution. Thus, traditional MCMC methods such as the Metropolis-Hastings algorithm, spend considerable amount of time of traversing space away from the mode of the target distribution. Our adapted MCMC algorithm aims in exploring the target distribution more efficiently.

The Metropolis-Hastings algorithm generates a Markov chain $\theta^{(1)}, \ldots, \theta^{(N)}$ which converges to a target distribution $\pi(\cdot)$ if N is large enough. In each update of the Markov chain, a candidate point, θ^* is sampled from a proposal density $q(\theta^* \mid \theta^{(i)})$, which gives the probability density of proposing θ^*, given the current, i^{th} value. With a certain probability or so-called acceptance ratio

$$a(\theta^*, \theta^{(i)}) = \min\left(1, \frac{q(\theta^* \mid \theta^{(i)})\pi(\theta^*)}{q(\theta^{(i)} \mid \theta^*)\pi(\theta^{(i)})}\right),$$

the proposed value is accepted.

The Metropolis algorithm is a special case of the Metropolis-Hastings algorithm where the proposal function is symmetrical. Since a symmetrical proposal distribution simplifies the calculation of the acceptance ratio to $a(\theta^*, \theta^{(i)}) = \min\left(1, \pi(\theta^*)/\pi(\theta^{(i)})\right)$, it is often used for updating parameters. The proposal function has a great influence on the speed of convergence and hence efficiency of the algorithm. We suggest a proposal distribution that speeds up the convergence towards the target distribution while limiting the additional computational effort. The idea behind our method is as follows: For each estimated parameter set θ there is a corresponding force of infection $\lambda(t)$ for each time t. It can be assumed that the mean force of infection $\overline{\lambda}$ is approximately constant over the number of iterations. The rationale behind it is that there is *true* mean force of infection that should be approximated by the MCMC algorithm. Proposing new parameter candidates depending on the mean force of infection reduces the volume that has to be traversed in order to converge to the target distribution. The resulting proposal density is not symmetric anymore and thus the procedure requires an adjustment of the acceptance ratio. The adapted Metropolis-Hastings algorithm to update the transmission parameters runs as follows:

<u>Two transmission routes</u>

1. Set initial values $\theta^{(0)} = \left(\alpha^{(0)}, \beta^{(0)}\right)$, and the number of iterations N.

2. Sample new parameter values α^*, β^* as follows:

   (a) Propose candidate α^* by sampling from $\alpha^{(i)} + \mathcal{N}(0, \sigma_\alpha^2)$

   (b) Propose candidate β^* assuming $\lambda^* \overset{!}{\approx} \lambda^{(i)} = \alpha^{(i)} + \beta^{(i)} \cdot \overline{Prev}$:
   Sample β^* from $\frac{\lambda^{(i)} - \alpha^*}{\overline{Prev}} + \mathcal{N}(0, \sigma_\beta^2)$, i.e. $\mathcal{N}\left(\frac{\lambda}{\overline{Prev}} - \frac{\alpha^*}{\overline{Prev}}, \frac{\sigma_\alpha^2}{\overline{Prev}^2} + \sigma_\beta^2\right)$

   (c) With probability

   $$\alpha(\theta^*, \theta^{(i)}) = \min\left(1, \frac{q(\theta^* \mid \theta^{(i)})\pi(\theta^*)}{q(\theta^{(i)} \mid \theta^*)\pi(\theta^{(i)})}\right)$$

   where $\frac{q(\theta^* \mid \theta^{(i)})}{q(\theta^{(i)} \mid \theta^*)} = e^{\frac{\beta^{*2} - \beta^{(i)2} + \mu_\beta(\beta^{(i)} - \beta^*)}{2\sigma_\beta^2}}$, accept the proposed value and set $\theta^{(i+1)} = \theta^*$, else set $\theta^{(i+1)} = \theta^{(i)}$.

3. If $i < N$, then go to step 2.

<u>Three transmission routes</u>

1. Set initial values $\theta^{(0)} = \left(\alpha^{(0)}, \beta^{(0)}, \epsilon^{(0)}, \mu^{(0)}\right)$, and the number of iterations N.

2. Sample new parameters $\theta^* = (\alpha^*, \beta^*, \epsilon^*, \mu^*)$ from a proposal density $q\left(\theta^* \mid \theta^{(i)}\right)$ as follows:

(a) Propose candidate α^* by sampling from $\alpha^{(i)} + \mathcal{N}(0, \sigma_\alpha^2)$

(b) Propose candidate β^* by sampling from $\beta^{(i)} + \mathcal{N}(0, \sigma_\beta^2)$

(c) Propose candidate μ_1 by sampling from $\mu^{(i)} + \mathcal{N}(0, \sigma_\mu^2)$

(d) Update $E_0^{(i+1)}$ to $\frac{\nu}{\mu^{(i)}} \cdot \overline{Prev}$

(e) Propose candidate ϵ^* assuming $\lambda^* \overset{!}{\approx} \lambda^{(i)} = \alpha^{(i)} + \beta^{(i)} \cdot \overline{Prev} + \epsilon^{(i)} \cdot E_0^{(i)}$:

Sample ϵ^* from $\frac{\lambda^{(i)} - \alpha^* - \beta^* \cdot \overline{Prev}}{E^{(i+1)}} + \mathcal{N}(0, \sigma_\epsilon^2)$,

i.e. $\mathcal{N}\left(\frac{\lambda^{(i)} - \alpha^* - \beta^* \cdot \overline{Prev}}{E^{(i+1}}, \frac{\sigma_\alpha^2}{E^{(i+1)^2}} + \frac{\sigma_\beta^2}{\left(\frac{E^{(i+1)}}{\overline{Prev}}\right)^2} + \sigma_\epsilon^2\right)$.

(f) With probability

$$\alpha(\theta^*, \theta^{(i)}) = \min\left(1, \frac{q(\theta^* \mid \theta^{(i)})\pi(\theta^*)}{q(\theta^{(i)} \mid \theta^*)\pi(\theta^{(i)})}\right)$$

where $\frac{q(\theta^* \mid \theta^{(i)})}{q(\theta^{(i)} \mid \theta^*)} = e^{\frac{\epsilon^{*2} - \epsilon^{(i)2} + 2\mu_\epsilon(\epsilon^{(i)} - \epsilon^*)}{2\sigma_\epsilon^2}}$, accept the proposed value and set $\theta^{(i+1)} = \theta^*$, else set $\theta^{(i+1)} = \theta^{(i)}$.

3. If $i < N$, then go to step 2.
