# Supplementary material for: Tracking Pseudomonas aeruginosa transmissions due to environmental contamination after discharge in ICUs using mathematical models
Source: PLoS Comput Biol. 2019 Aug 28;15(8):e1006697. doi: 10.1371/journal.pcbi.1006697 (PMC6736315; doi:10.1371/journal.pcbi.1006697)
Supplement: S6 Text — (PDF) [file pcbi.1006697.s006.pdf]

S6 Text. Model selection. We would like to assess whether we can concatenate the Besançon data e.g. before and after the renovation of the ICUs in one large data set to increase the power of our method. The idea is to compare the DICs for two different scenarios:

- Consider only one model including one parameter set $\theta = \{\alpha, \beta, f, \phi\}$ where α is the endogenous, β the cross-transmission parameter, f the importation rate and ϕ the test sensitvity. The analysis is then performed on all the data X of the two ICUs and the two time periods (before and after renovation). The DIC is then given by

$$\mathrm{DIC}_{X,\theta} = \overline{D_X(\theta)} + \frac{1}{2}\mathrm{Var}(D_X(\theta)).$$

- Consider a model including a parameter set consisting of separate parameters for each time period:
  - $\theta_1 = \{\alpha_1, \beta_1, f_1, \phi_1\}$
  - $\theta_2 = \{\alpha_2, \beta_2, f_2, \phi_2\}$

  The parameter set of the model is then:

$$\theta = \theta_1 \cup \theta_2 = \{\alpha_1, \beta_1, f_1, \phi_1, \alpha_2, \beta_2, f_2, \phi_2\}.$$

  The parameters in θ_1 are updated for the data set before renovation whereas the parameters in θ_2 are updated for the data set after renovation. The deviance for this model is determined by

$$\begin{aligned} D_X(\theta) &= -2\log\pi(X \mid \theta) \\ &= -2\log\pi(\{X_1, X_2\} \mid \theta_1 \cup \theta_2) \\ &= -2\log\left[\pi(X_1 \mid \theta_1 \cup \theta_2) \cdot \pi(X_2 \mid \theta_1 \cup \theta_2)\right] \\ &= -2\log\left[\pi(X_1 \mid \theta_1) \cdot \pi(X_2 \mid \theta_2)\right] \\ &= -2\left[\log\pi(X_1 \mid \theta_1) + \log\pi(X_2 \mid \theta_2)\right] \\ &= D_{X_1}(\theta_1) + D_{X_2}(\theta_2), \end{aligned}$$

  where X_1 is the data set for the time period before and X_2 for the time period after renovation. Thus, the DIC for the model including separate parameters for each time period can be calculated as

$$\mathrm{DIC}_{X,\theta} = \mathrm{DIC}_{X_1,\theta_1} + \mathrm{DIC}_{X_2,\theta_2}.$$
