# Supplementary material for: Tracking Pseudomonas aeruginosa transmissions due to environmental contamination after discharge in ICUs using mathematical models
Source: PLoS Comput Biol. 2019 Aug 28;15(8):e1006697. doi: 10.1371/journal.pcbi.1006697 (PMC6736315; doi:10.1371/journal.pcbi.1006697)
Supplement: S7 Text — (PDF) [file pcbi.1006697.s007.pdf]

S7 Text. Model assessment. To assess our method's ability to detect large discrepancies between the data and model assumptions, we simulated a data set with a substantial contribution of background, cross-transmission and environmental contamination after discharge ($27\%, 24\%$ and 49%). We analyzed the data set with our MCMC procedure including only background transmission as a transmission route. Thus, the model in the MCMC process assumed a constant force of infection. In Fig S34, we can see that the expected coverage probabilities are not met. Hence, it can be asserted that there is a large discrepancy between the model and the data. The data can be found on `https://github.com/tm-pham/transmissionPA`.
