# Supplementary material for: Tracking Pseudomonas aeruginosa transmissions due to environmental contamination after discharge in ICUs using mathematical models
Source: PLoS Comput Biol. 2019 Aug 28;15(8):e1006697. doi: 10.1371/journal.pcbi.1006697 (PMC6736315; doi:10.1371/journal.pcbi.1006697)
Supplement: S8 Text — (PDF) [file pcbi.1006697.s008.pdf]

S8 Text. Prior distributions. Sensitivity analyses were performed using different priors. We performed the main analyses of the Besançon (as presented in the *Results* section) with uninformative exponential priors and small intitial values for the standard deviation of the proposal distribution.

Further analyses were performed using a weakly informative exponential prior Exp(1.0) and a uniform prior $\mathrm{U}(0,2)$ for the decay rate μ. The histograms and traceplots using Exp(1.0) show that these results are not different from results using an uninformative exponential prior. However, for the uniform prior $U(0,2)$, the MCMC chain shows signs of non-convergence. The values for μ have a strong tendency towards the upper boundary and a strong correlation with ϵ (see S33). This behaviour was also observed when the full model was applied to simulated data sets with no environmental contamination after discharge (see S9) and can be explained as follows: A scenario with no environmental contamination after discharge is indistinguishable from a scenario with environmental contamination but very short bacterial persistence in the environment (i.e. high values of μ). In such a case, several combinations of ϵ and μ and β reflect the same situation. In particular, any high value of μ may reflect the absence of environmental contamination. The results of our sensitivity analyses confirm that for the two data sets of the Besançon hospital, environmental contamination after discharge is only of minor influence. Further elaborations on the influence of different values of the decay rate μ can be found in S9.
