# Supplementary material for: Tracking Pseudomonas aeruginosa transmissions due to environmental contamination after discharge in ICUs using mathematical models
Source: PLoS Comput Biol. 2019 Aug 28;15(8):e1006697. doi: 10.1371/journal.pcbi.1006697 (PMC6736315; doi:10.1371/journal.pcbi.1006697)
Supplement: S9 Text — (PDF) [file pcbi.1006697.s009.pdf]

S9 Text. Simulation studies. Several data sets were simulated to test the algorithm's ability to identify the correct relative contribution. We performed two types of simulations:

1. The duration of persistence of bacterial load is varied.
2. The relative contribution of envionmental contamination after discharge is varied.

For the first simulation study, we fixed values for α, β and ν and varied values for μ. The parameter ϵ is set to $0.6 \cdot \mu$. Three main scenarios regarding the duration of persistence of bacterial load in the environment are analyzed:

1. Long: $\mu \in \{1/7, 1/14\}$
2. Medium: $\mu \in \{1, 1/3\}$
3. Short: $\mu \in \{2, 5\}$

For each of the three scenarios the importation rate is varied within $\{0.01, 0.05, 0.1\}$.

We observed that chain convergence cannot be attained in a reasonable amount of time using uninformative exponential priors. For medium duration of bacterial persistence convergence could be achieved using either a weakly informative prior Exp(1.0) and a uniform prior $\mathrm{U}(0, 2)$. The reasons for non-convergence for long or short bacterial persistence in the environment as well as the justification of the weakly informative and uniform prior are based on the same reasoning.

If bacterial persistence is set to be long (longer than the average length of stay of patients), then environmental contamination after discharge stays approximately at one level and the resulting probability of colonization due to this route is approximately constant. Thus, the induced fluctuations in the prevalence can be hardly distinguished from fluctuations due to background transmission. On the other hand, a short duration of bacterial persistence (much shorter than the average length of stay) leads to difficulties in distinguishing the resulting model from one with a higher contribution of cross-transmission and smaller contribution of environmental contamination. Hence, based on the fluctuations of the prevalence, only a medium length of bacterial persistence is meaningful and the restriction of the parameter space or the use of more informative priors is justified.

For the second simulation, we varied the relative contribution of environmental contamination after discharge. In particular, when environmental contamination after discharge was not present in the simulated data, the results resembled our analysis of the Besançon data shown in the *Results* section.

The histograms and plots corresponding to the described simulation studies can be found on `https://github.com/tm-pham/transmissionPA`. Further information on our simulation studies may be requested from the first author.
