# Supplementary material for: Tracking Pseudomonas aeruginosa transmissions due to environmental contamination after discharge in ICUs using mathematical models
Source: PLoS Comput Biol. 2019 Aug 28;15(8):e1006697. doi: 10.1371/journal.pcbi.1006697 (PMC6736315; doi:10.1371/journal.pcbi.1006697)
Supplement: S10 Text — (PDF) [file pcbi.1006697.s010.pdf]

**S10 Text. Secondary analyses.** In addition to the analyses presented in *Results*, six further analyses were performed. For each ICU, the time periods before and after renovation were combined. Finally, all available data was concatenated into one big data set and analyzed at once. The results of these analyses using the submodel as well as the full model are presented in S12 - S13 Tables. The posterior estimates of the model parameters and the corresponding relative contributions are similar to the ones presented in the *Results* section.
