# Supplementary material for: Tracking Pseudomonas aeruginosa transmissions due to environmental contamination after discharge in ICUs using mathematical models
Source: PLoS Comput Biol. 2019 Aug 28;15(8):e1006697. doi: 10.1371/journal.pcbi.1006697 (PMC6736315; doi:10.1371/journal.pcbi.1006697)
Supplement: S11 Text — (PDF) [file pcbi.1006697.s011.pdf]

S11 Text. Impact of prior colonized bed occupants. In a first step, we performed Fisher's exact test to study the association between colonization status of current and prior bed occupants for the data sets of the University Hospital of Besançon. The 2×2 tables can be found in S14 and S15.

Interestingly, there is a significant association for ICU B (OR: 1.54, 95% cI: (1.24, 1.91), p-value: 0.0001) but none for ICU A (OR: 0.95, 95% cI: (0.7, 1.27), p-value: 0.7707). However, such a simple test may be confounded. Consecutive colonized patients may be a result due to cross-transmission rather than an increased risk of prior bed occupants.

In order to disentangle the effects of cross-transmission and prior bed occupants, we analyzed the data sets of the University Hospital of Besançon using the following simple model: Each bed occupant i faces a force of infection λ_i depending on the colonization status of the prior bed occupant:

$$\lambda_i(t) = \alpha + \beta \frac{I(t)}{N(t)} + p \cdot \mathbb{1}(c_i^{\text{prior}}) \tag{7}$$

where c_i^{prior} is the colonization status of the prior bed occupant and $\mathbb{1}(c_i^{\text{prior}}) = 1$ if $c_i^{\text{prior}} = 1$ and 0 otherwise. It represents the increased risk that patients experience when occupying a room/bed of a prior colonized bed occupant. The parameter estimates and the relative contributions can be found in Table S16.

The results show that the influence of prior bed occupants is only limited ($< 6\%$) for both ICUs of the University Hospital of Besançon. Simulation studies confirm that a significant impact of this route would be detected by this model. The code of the MCMC procedure for this analysis, the histogram and traceplots for the simulations studies and the data sets of Besançon can be found on `https://github.com/tm-pham/transmissionPA`.
