# Supplementary material for: Tracking Pseudomonas aeruginosa transmissions due to environmental contamination after discharge in ICUs using mathematical models
Source: PLoS Comput Biol. 2019 Aug 28;15(8):e1006697. doi: 10.1371/journal.pcbi.1006697 (PMC6736315; doi:10.1371/journal.pcbi.1006697)
Supplement: S12 Text — (PDF) [file pcbi.1006697.s012.pdf]

S12 Text. COMBACTE-MAGNET membership list. Please find below the list of COMBACTE-MAGNET consortium partners:

Albert Vermaas, Alex Waehry, Angela Supplitt, Anne Adams, Antonio Portolés Pérez, Aurore Drecq, Brian Allen, Christophe Misse, Cornelia Mockwitz, Fanny Senez, Felicity Jane Gabbay, Freek De Jong, Gabriella Monaco, Gill Wells, Heather Rogers, Henrik Landstrom, Hermann Hayn, Holger Schmoll, Jaime Caro Aguirre, Jantine Spithoven, Josep M. Campistol, Juergen Dreyer, Karine Clement, Karl-Heinz Müller, Lauren Fleming, Lea Pais, Lynsey Keig, Malcolm Skingle, Maria Carol Sanjurjo, Marion Do Maria, Markus Jäger, Michael Browne, Nicola Williams, Pascal Savary, Patricia Gizecki, Renaud Mazy, Sarah Everett-Cox, Sofia Karakostas, Tommaso Rupolo, Ursula Theuretzbacher, Virginia Nieto Guerrero, Wilfried Reincke, Alain Verschoren, Alexander Affeldt, Alfredo García Díaz, Andreas Rothfuss, Carlo Giaquinto, Christine Clerici, Daniel Wyler, Denis Hochstrasser, Dieter Kaufmann, Dirkjan Masman, Frank Miedema, Helen Steel, Holger Zimmermann, Jaap Verweij, Jan-Olof Jacke, Jean-François Lefebre, John Graham, José Francisco Soto Bonel, Jose Manuel Aranda Lara, Josep M. Campistol Plana, Laurence Lomme, Marcel Levi, María Dolores Acón, Markus Müller, Maya Saïd, Nicola Sartor, Noureddine Farah, Pastora Martinez Samper, Pierre-François Leyvraz, Renaud Mazy, Ron Scott, Yves Geysels, Andreas Kümin, Anthony Latte, Clemens Lässing, Elena Ferragut Roig, Eleonora Zuolo, Esther Bettiol, Eva Lindgren, Eveline Bielser, Gülseren Yalvac, Jenny Lawson, José Ángel Freire Astray, Jose Soto Bonel, Julia Lloyd-Parks, Jürgen Dreyer, Malgorzata Kielbasa, Marco Perdon, Markus Zeitlinger, Michaela Schuhmacher, Michiel Gerlagh, Olivier Brun, Pam Neagle, Patricia Schott, Rebecca Smith, Sally Miles, Sophie Monteau, Susanna Montalto, Thierry Borloz, Wouter Roobol, Xavier Fretille, Abdel Oualim, Alasdair Macgowan, Andreas Voss, Andrew Lovering, Anne Witschi, Antoni Torres, Antonio Oliver, Bruno Francois, Craig Maclean, Cuong Vuong, David Evans, Evelina Tacconelli, Hasan Jafri, Ingrid Klingmann, Jan Beyersmann, Jean Chastre, Jean-François Timsit, Jesús Rodriguez Baño, Johan Mouton, Kim Gilchrist, Leonard Leibovici, Leonhard Held, Marc Bonten, Martin Wolkewitz, Mervyn Singer, Miguel Sanchez, Mike Sharland, Miquel Pujol Rojo, Philippe Eggimann, Philippe Montravers, Pierre-François Laterre, Richard Bax, Richard Fitzgerald, Stephan Harbarth, Surbhi Malhotra-Kumar, Tom van der Poll, William Hope.
