# Supplementary material for: Tracking Pseudomonas aeruginosa transmissions due to environmental contamination after discharge in ICUs using mathematical models
Source: PLoS Comput Biol. 2019 Aug 28;15(8):e1006697. doi: 10.1371/journal.pcbi.1006697 (PMC6736315; doi:10.1371/journal.pcbi.1006697)
Supplement: S1 Table — (PDF) [file pcbi.1006697.s013.pdf]

S1 Table. Summary statistics of the marginal posterior distributions for parameters of the submodel based on the analysis of the Besançon data.

Parameter	Symbol	Median (95% credibility interval)*		
		ICU A	ICU B	ICUs combined
Background coefficient	α	0.011 (0.008, 0.015)	0.008 (0.006, 0.011)	0.009 (0.007, 0.011)
Cross-transmission coefficient	β	0.03 (0.012, 0.046)	0.038 (0.006, 0.011)	0.034 (0.024, 0.044)
Sensitivity	ϕ (%)	50.7 (48.0, 53.2)	60.5 (58.8, 62.1)	57.5 (56.2, 58.9)
Importation probability	f (%)	5.5 (4.5, 6.5)	7.6 (6.7, 8.6)	6.5 (5.8, 7.2)
Fraction colonized	p_{col} (%)	22.2 (21.2, 23.1)	23.2 (22.7, 23.7)	22.4 (21.9, 22.8)
**Contributions**				
Background contribution	$R_{\text{background}}$ (%)	65.1 (46.4, 84.6)	51.1 (35.5, 66.9)	57.6 (46.0, 68.9)
Cross-transmission contribution	R_{crossT} (%)	34.9 (15.4, 53.6)	48.9 (33.1, 64.5)	42.4 (31.1, 54.0)

*Highest posterior density interval
