# Supplementary material for: Tracking Pseudomonas aeruginosa transmissions due to environmental contamination after discharge in ICUs using mathematical models
Source: PLoS Comput Biol. 2019 Aug 28;15(8):e1006697. doi: 10.1371/journal.pcbi.1006697 (PMC6736315; doi:10.1371/journal.pcbi.1006697)
Supplement: S2 Table — (PDF) [file pcbi.1006697.s014.pdf]

**S2 Table. Summary statistics of the marginal posterior distributions for parameters of the full model based on the analysis the Besançon data.**

| Parameter | Symbol | Median (95% credibility interval)* | | | | | |
|---|---|---|---|---|---|---|---|
| | | ICU A | | ICU B | | ICUs combined | |
| Background coefficient | $\alpha$ | 0.011 | $(0.008, 0.014)$ | 0.008 | $(0.006, 0.011)$ | 0.009 | $(0.007, 0.011)$ |
| Cross-transmission coefficient | $\beta$ | 0.013 | $(0, 0.033)$ | 0.022 | $(0, 0.011)$ | 0.018 | $(0, 0.034)$ |
| Environmental coefficient | $\epsilon$ | 201.1 | $(0.018, 832.829)$ | 176.7 | $(0.009, 811.6)$ | 209.0 | $(0.02, 784.5)$ |
| Decay rate | $\mu$ | 1415.8 | $(65.6, 4273.8)$ | 1396.9 | $(26.4, 3992.2)$ | 1419.2 | $(43.7, 4524.0)$ |
| Sensitivity | $\phi\,(\%)$ | 50.6 | $(48.0, 53.1)$ | 60.5 | $(58.9, 62.1)$ | 57.6 | $(56.2, 58.9)$ |
| Importation probability | $f\,(\%)$ | 5.5 | $(4.5, 6.6)$ | 7.5 | $(6.6, 8.4)$ | 6.4 | $(5.7, 7.2)$ |
| Fraction colonized | $p_{\text{col}}\,(\%)$ | 22.2 | $(21.3, 23.1)$ | 23.2 | $(22.7, 23.6)$ | 22.4 | $(21.9, 22.8)$ |
| **Contributions** | | | | | | | |
| Background | $R_{\text{background}}\,(\%)$ | 62.2 | $(44.8, 79.7)$ | 50.4 | $(35.6, 65.2)$ | 57.6 | $(45.5, 67.9)$ |
| Cross-transmission | $R_{\text{crossT}}\,(\%)$ | 37.2 | $(20.4, 54.9)$ | 49.0 | $(34.0, 63.1)$ | 42.7 | $(31.9, 54.0)$ |
| Env. cont. after discharge | $R_{\text{env}}\,(\%)$ | 0.006 | $(0, 0.013)$ | 0.005 | $(0, 0.013)$ | 0.006 | $(0, 0.012)$ |

*Highest posterior density interval
