# Supplementary material for: Tracking Pseudomonas aeruginosa transmissions due to environmental contamination after discharge in ICUs using mathematical models
Source: PLoS Comput Biol. 2019 Aug 28;15(8):e1006697. doi: 10.1371/journal.pcbi.1006697 (PMC6736315; doi:10.1371/journal.pcbi.1006697)
Supplement: S3 Table — (PDF) [file pcbi.1006697.s015.pdf]

S3 Table. Association of colonization statuses of consecutive bed occupants in ICU A of the University Hospital of Besançon.

		Current bed occupant	
		Colonized	Non-colonized
Prior bed occupant	Colonized	57	478
	Non-colonized	481	3826
