# Supplementary material for: Tracking Pseudomonas aeruginosa transmissions due to environmental contamination after discharge in ICUs using mathematical models
Source: PLoS Comput Biol. 2019 Aug 28;15(8):e1006697. doi: 10.1371/journal.pcbi.1006697 (PMC6736315; doi:10.1371/journal.pcbi.1006697)
Supplement: S4 Table — (PDF) [file pcbi.1006697.s016.pdf]

S4 Table. Association of colonization statuses of consecutive bed occupants in ICU B of the University Hospital of Besançon.

		Current bed occupant	
		Colonized	Non-colonized
Prior bed occupant	Colonized	123	586
	Non-colonized	610	4479
