# Supplementary material for: Tracking Pseudomonas aeruginosa transmissions due to environmental contamination after discharge in ICUs using mathematical models
Source: PLoS Comput Biol. 2019 Aug 28;15(8):e1006697. doi: 10.1371/journal.pcbi.1006697 (PMC6736315; doi:10.1371/journal.pcbi.1006697)
Supplement: S5 Table — (PDF) [file pcbi.1006697.s017.pdf]

S5 Table. Summary statistics of the marginal posterior distributions for parameters of model (7) based on the analysis of the Besançon data.

Parameter	Symbol	Median (95% credibility interval)*			
		ICU A		ICU B	
Background coefficient	α	0.011	$(0.008, 0.014)$	0.008	$(0.006, 0.011)$
Cross-transmission coefficient	β	0.027	$(0.013, 0.041)$	0.036	$(0.023, 0.048)$
Prior bed occupant coefficient	p	0.004	$(0, 0.007)$	0.003	$(0, 0.006)$
Sensitivity	$\phi\,(\%)$	50.6	$(48.0, 53.1)$	60.5	$(58.9, 62.1)$
Importation probability	$f\,(\%)$	5.5	$(4.5, 6.6)$	7.5	$(6.6, 8.4)$
Fraction colonized	$p_{\mathrm{col}}\,(\%)$	22.2	$(21.3, 23.1)$	23.2	$(22.7, 23.6)$
**Contributions**					
Background	$R_{\mathrm{background}}\,(\%)$	65.1	$(48.8, 81.7)$	50.4	$(35.7, 66.6)$
Cross-transmission	$R_{\mathrm{crossT}}\,(\%)$	31.8	$(14.6, 47.2)$	46.8	$(31.3, 62.1)$
Prior bed occupants discharge	$R_{\mathrm{prior}}\,(\%)$	3.1	$(0, 6)$	2.6	$(0, 5.6)$

*Highest posterior density interval
