# Supplementary figures and images for: Tracking Pseudomonas aeruginosa transmissions due to environmental contamination after discharge in ICUs using mathematical models
Source: PLoS Comput Biol. 2019 Aug 28;15(8):e1006697. doi: 10.1371/journal.pcbi.1006697 (PMC6736315; doi:10.1371/journal.pcbi.1006697)

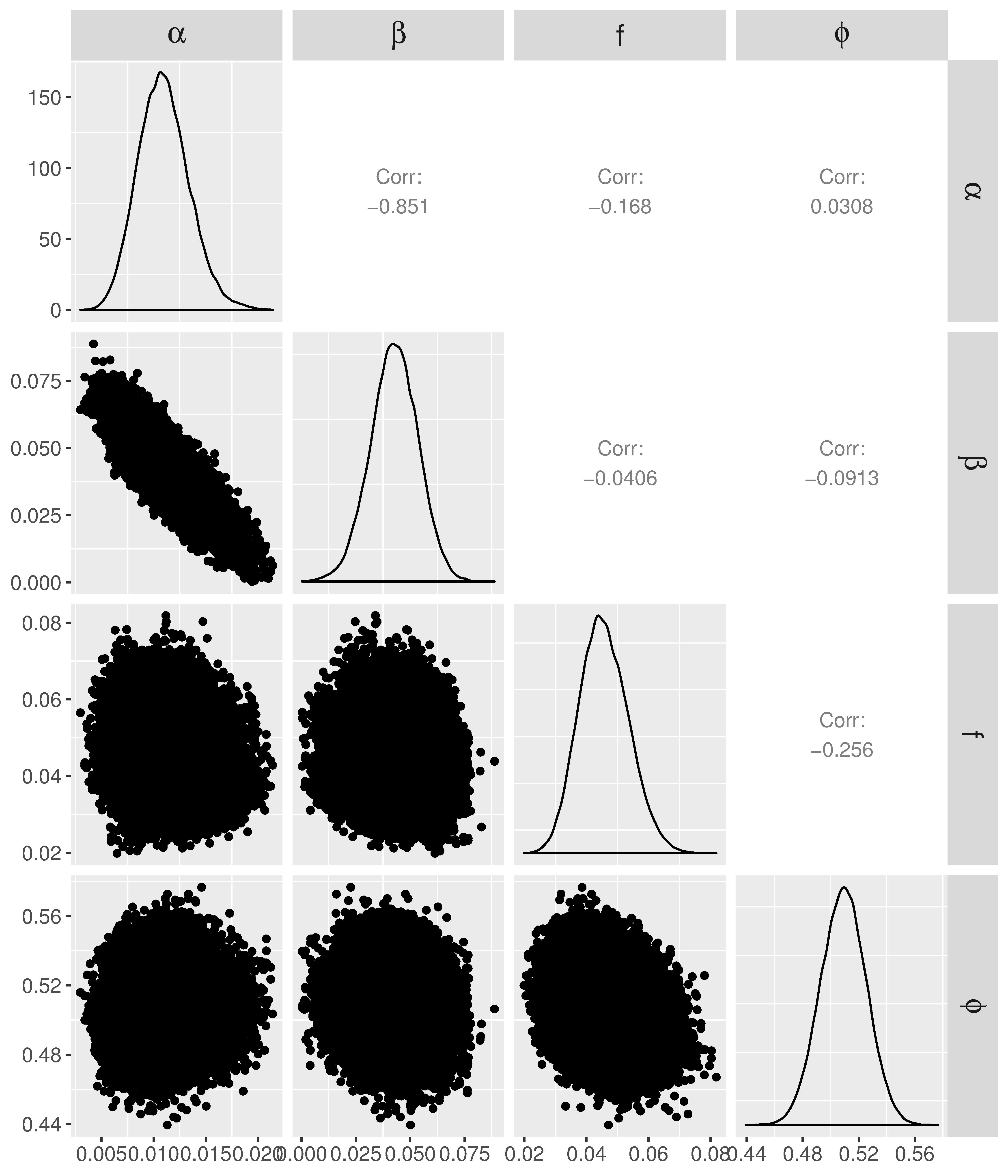

Supplement: S1 Fig — The plots were generated from the data of ICU A before renovation using the submodel with background and cross-transmission. (TIF) [file pcbi.1006697.s018.tif]

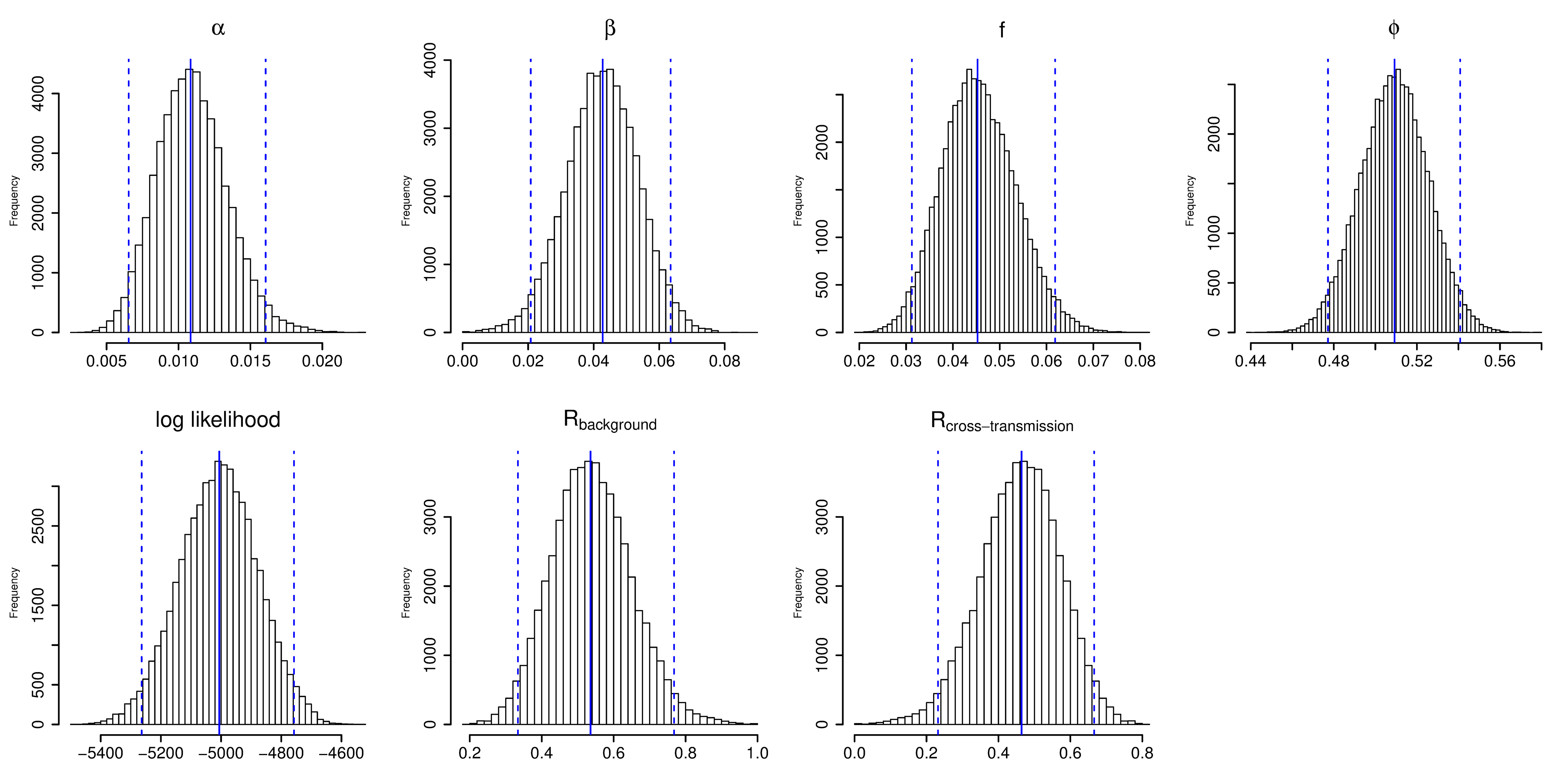

Supplement: S2 Fig — (TIF) [file pcbi.1006697.s019.tif]

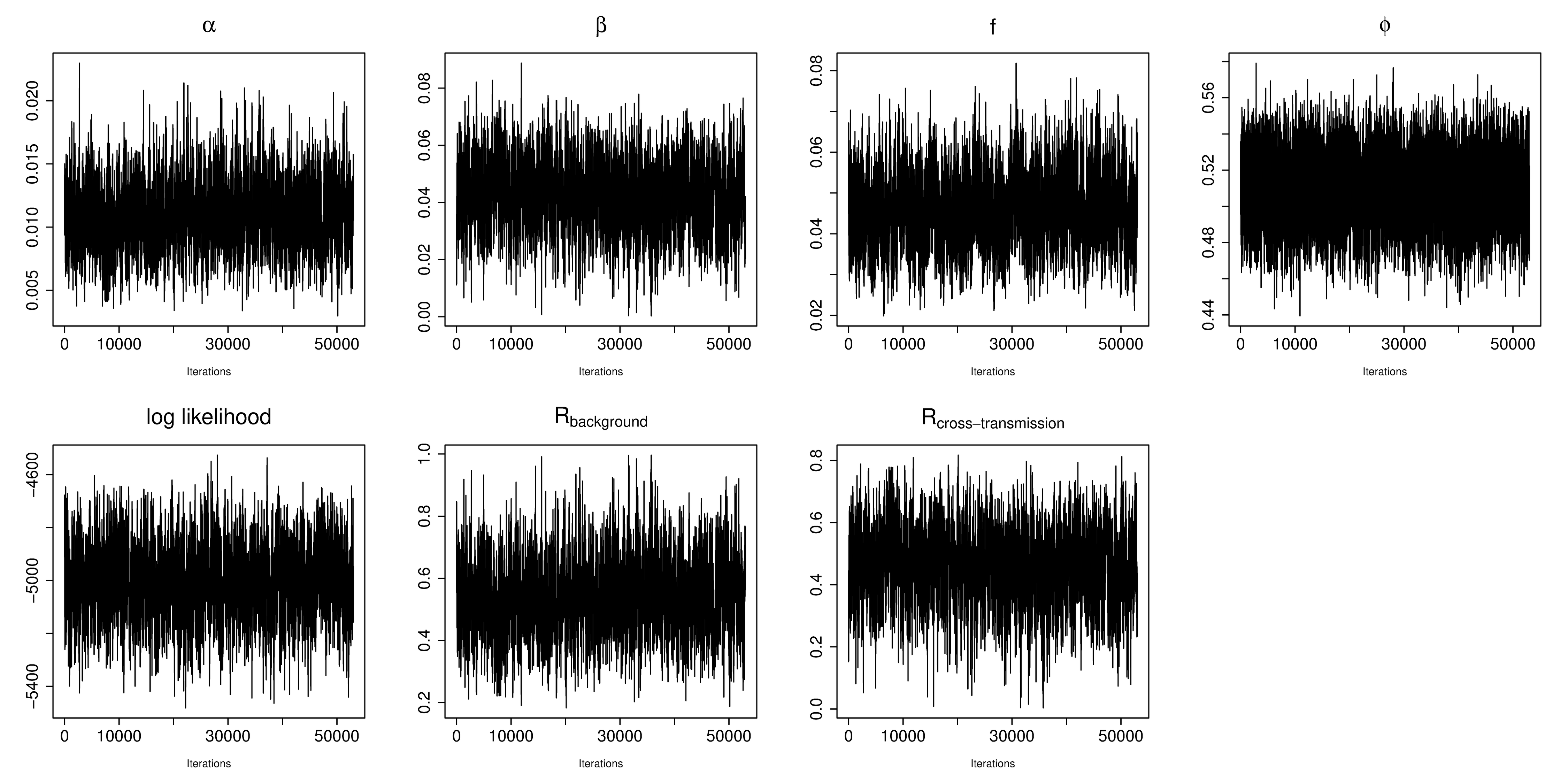

Supplement: S3 Fig — (TIF) [file pcbi.1006697.s020.tif]

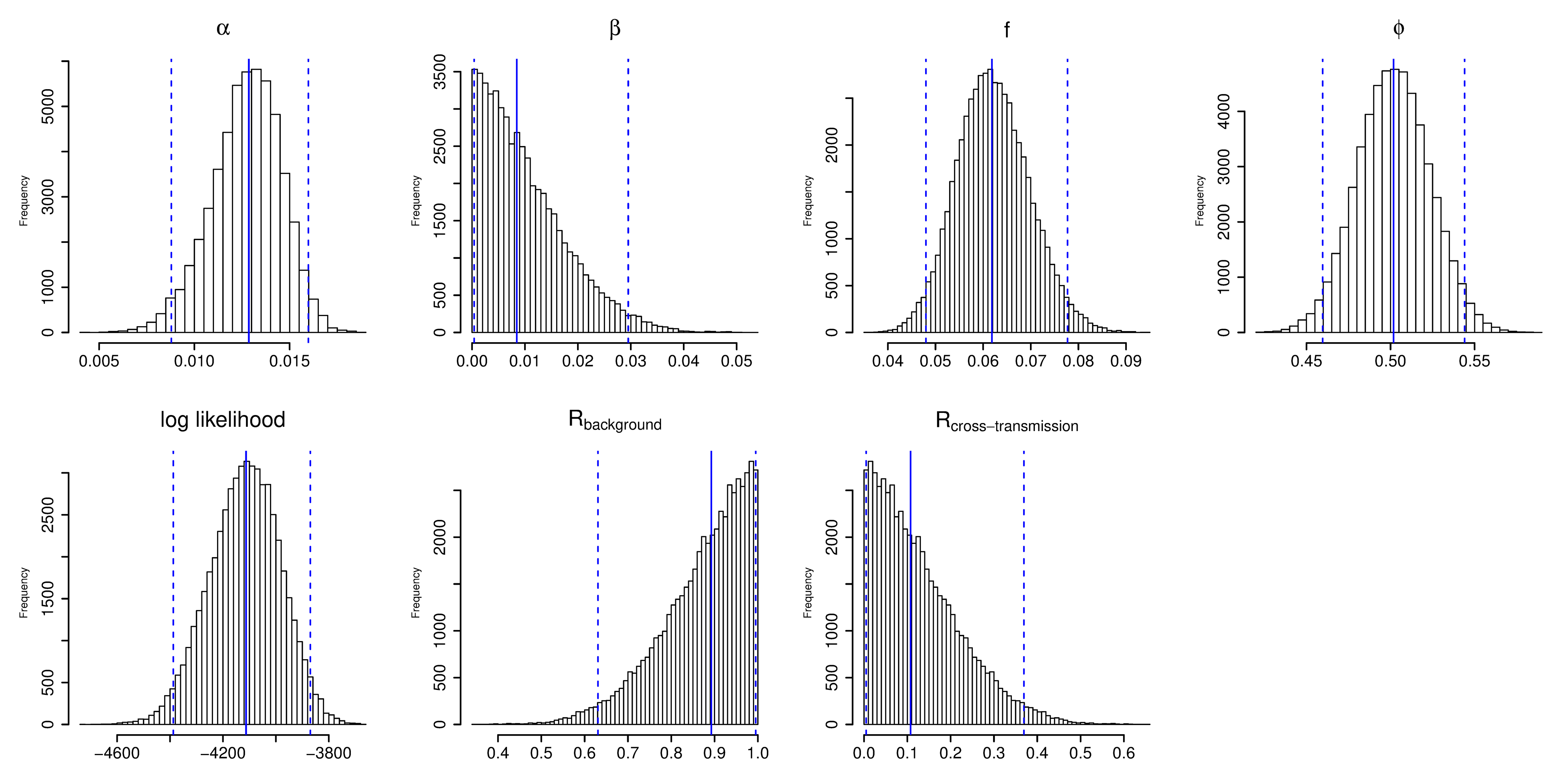

Supplement: S4 Fig — (TIF) [file pcbi.1006697.s021.tif]

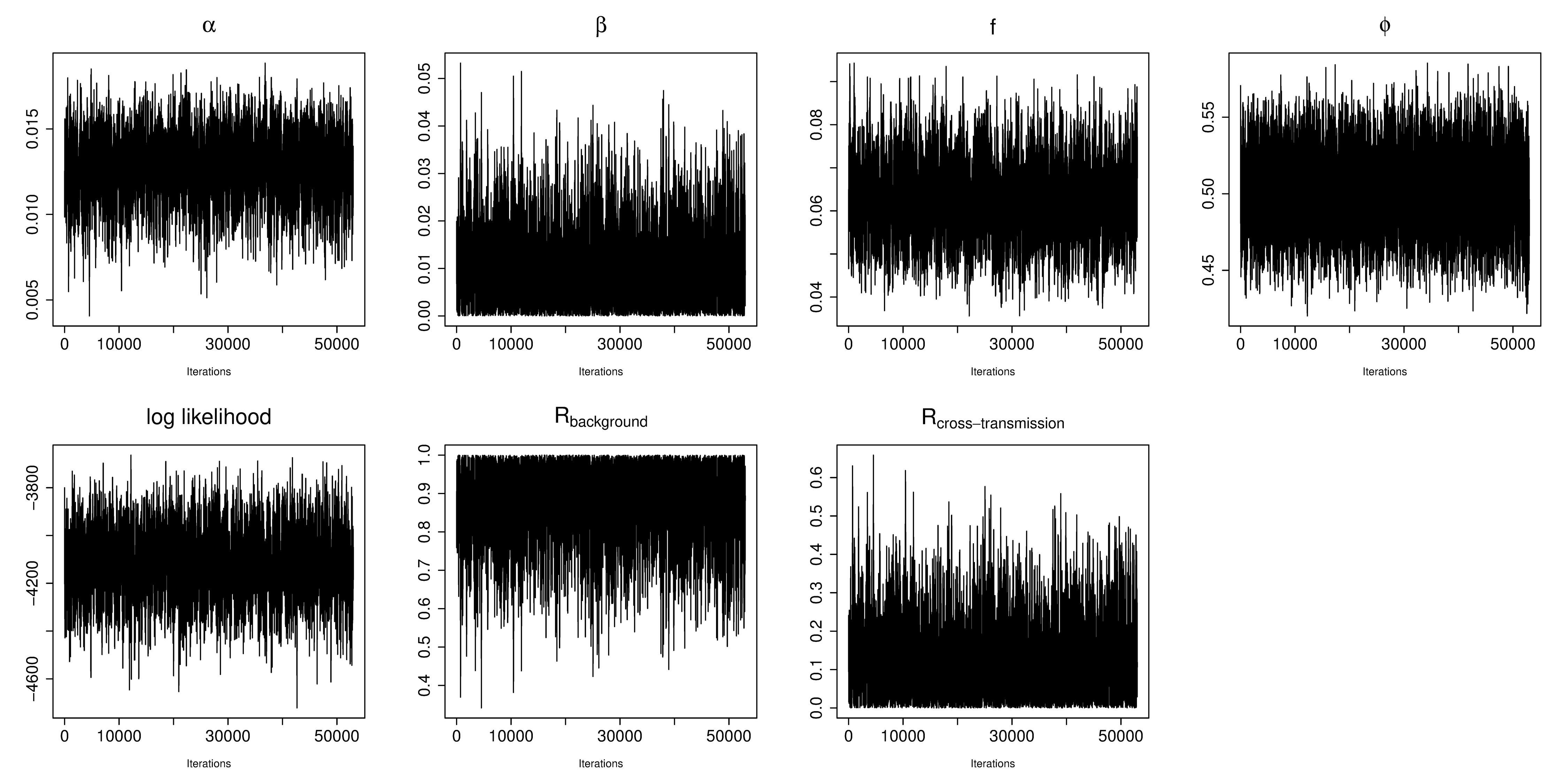

Supplement: S5 Fig — (TIF) [file pcbi.1006697.s022.tif]

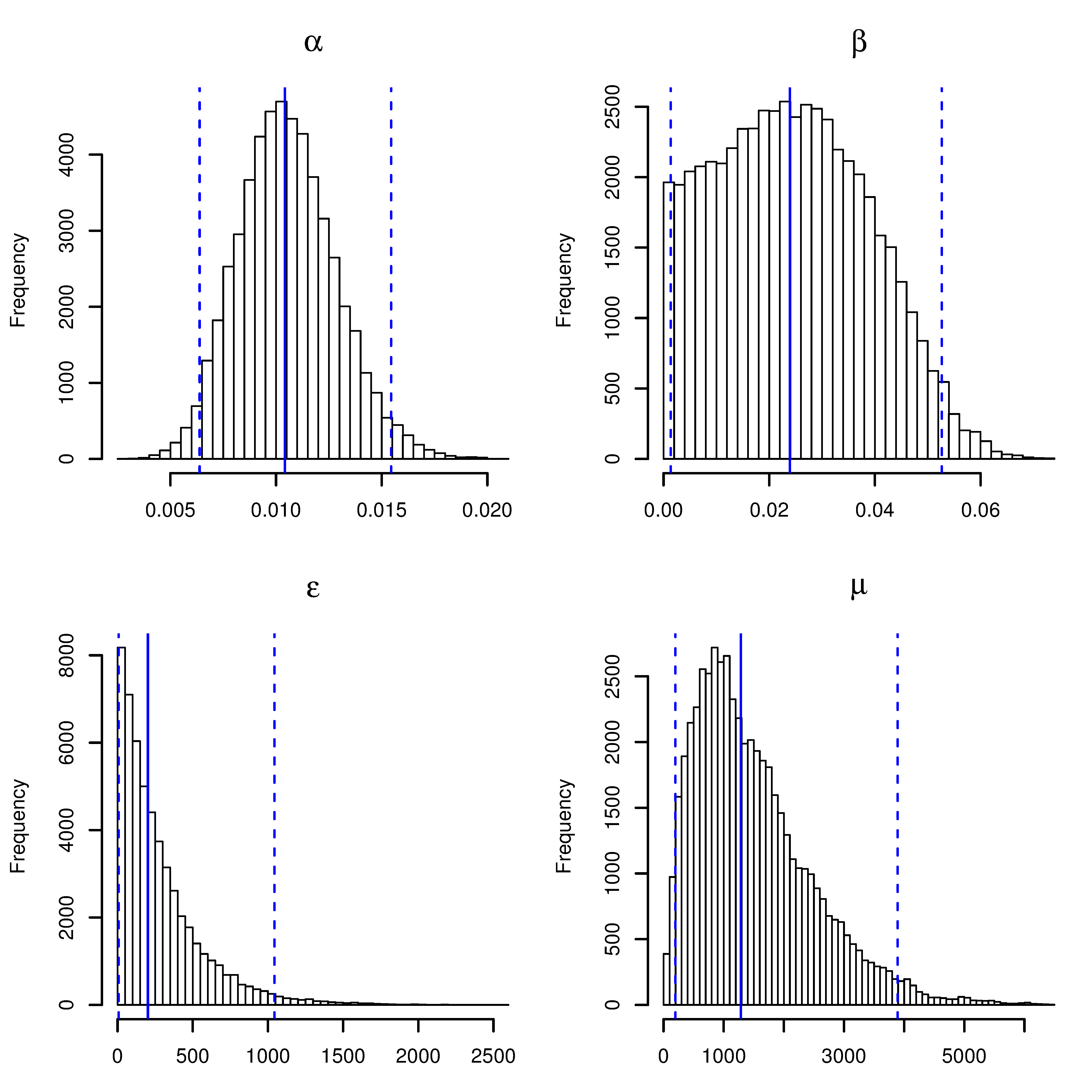

Supplement: S6 Fig — The results are displayed for transmission parameters α, β, ϵ and μ. (TIF) [file pcbi.1006697.s023.tif]

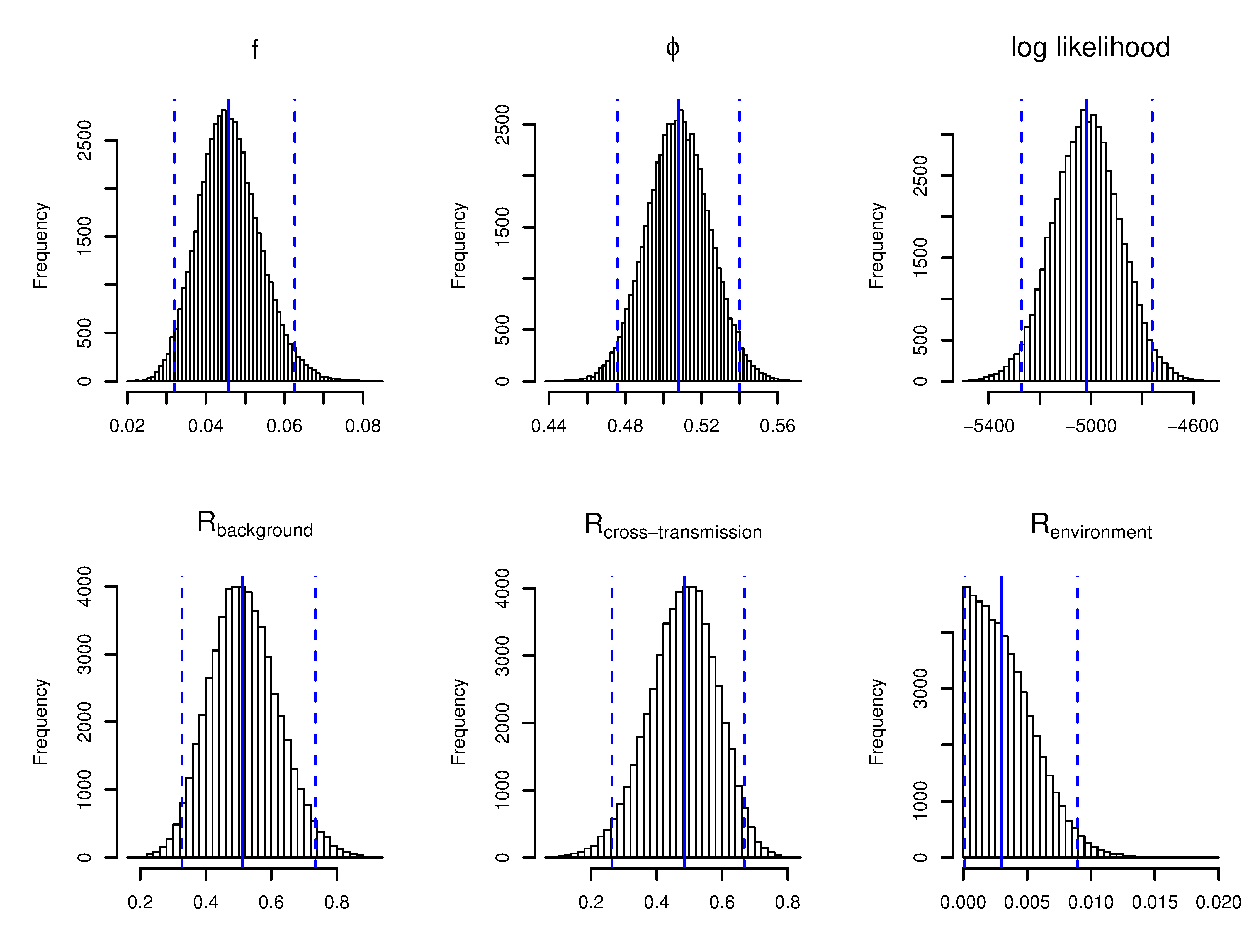

Supplement: S7 Fig — The results are displayed for the importation probability f, sensitivity parameter ϕ, relative contributions Ri, i ∈ {background, cross- transmission, environment} and log-likelihood. (TIF) [file pcbi.1006697.s024.tif]

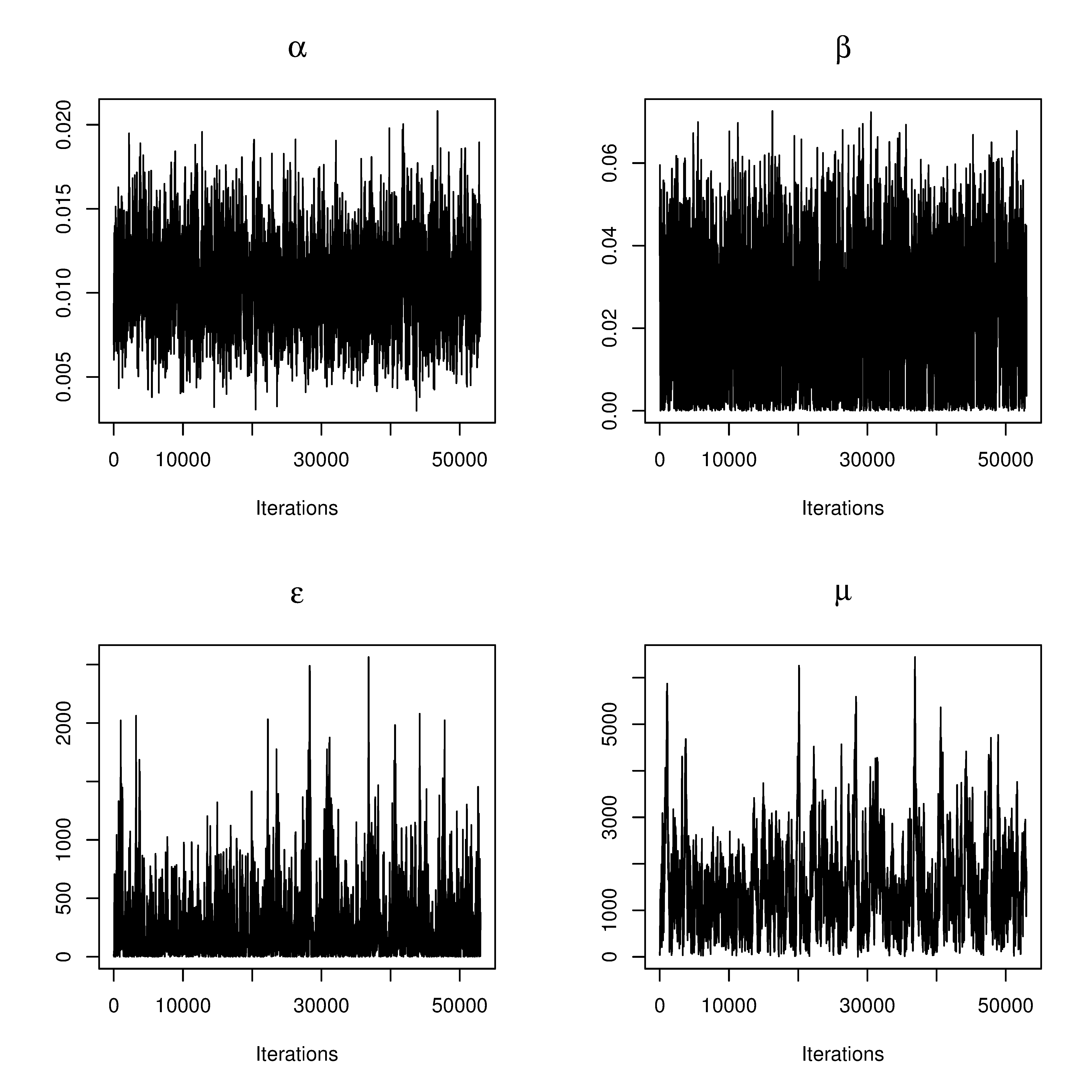

Supplement: S8 Fig — The results are displayed for transmission parameters α, β, ϵ and μ. (TIF) [file pcbi.1006697.s025.tif]

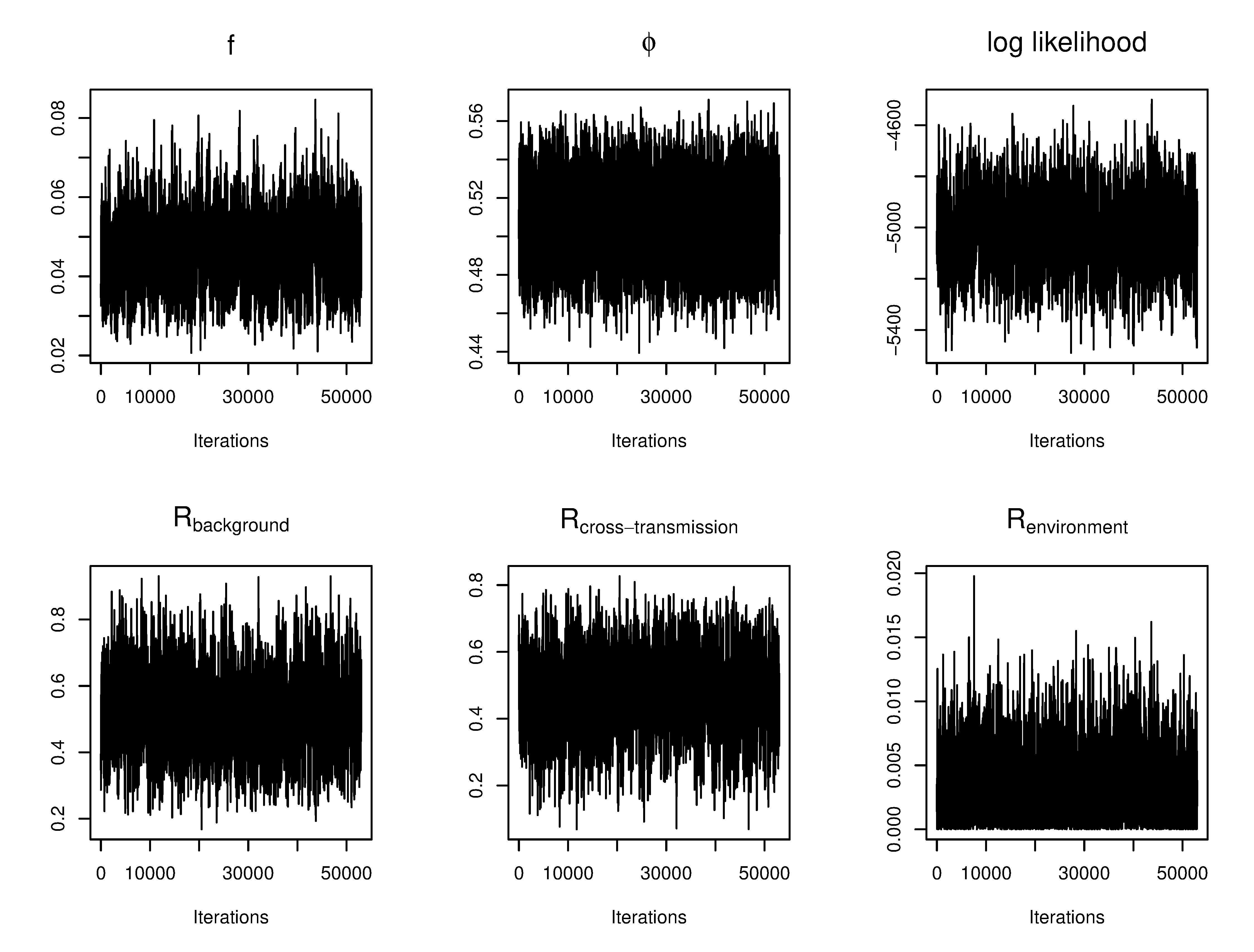

Supplement: S9 Fig — The results are displayed for the importation probability f, sensitivity parameter ϕ, relative contributions Ri, i ∈ {background, cross- transmission, environment} and log-likelihood. (TIF) [file pcbi.1006697.s026.tif]

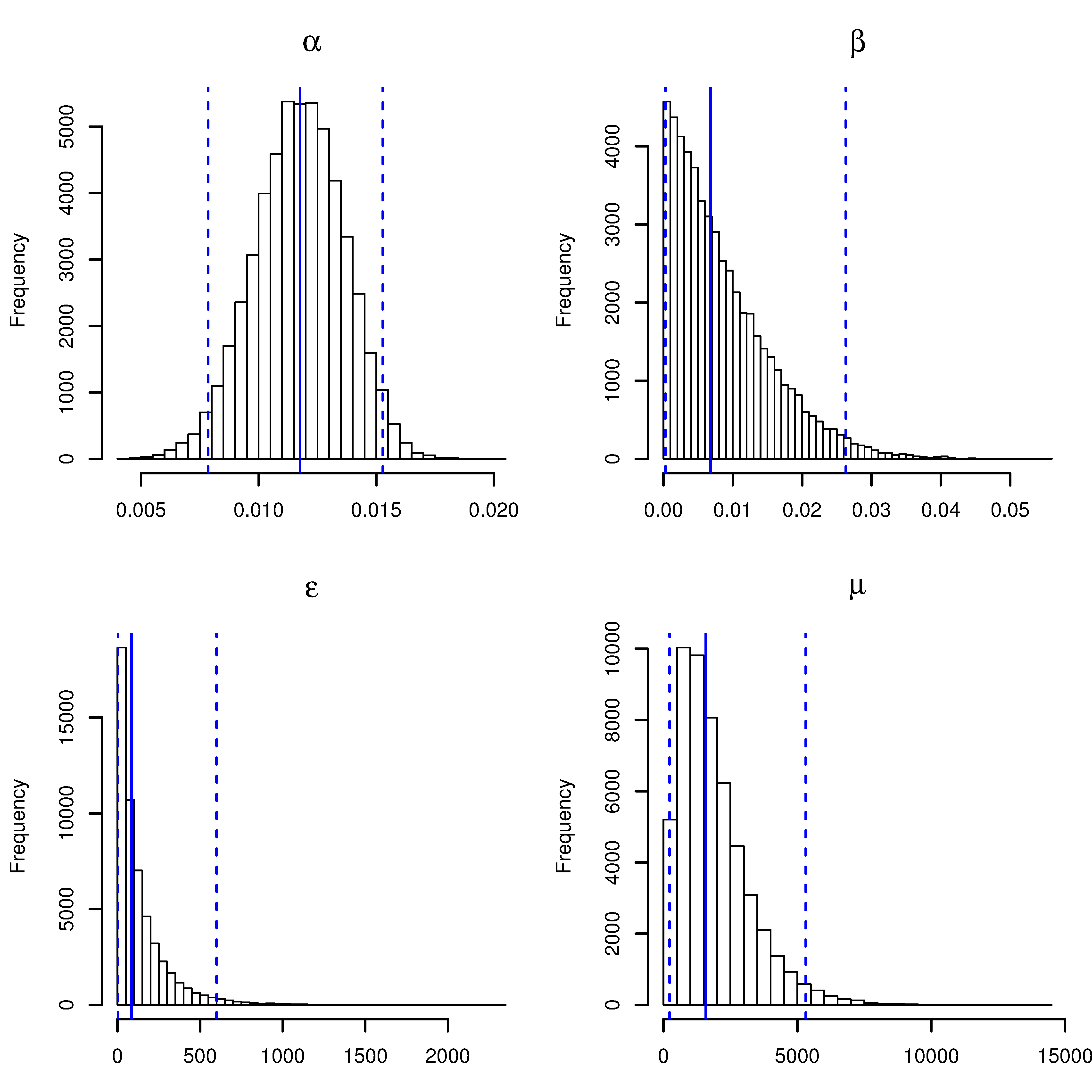

Supplement: S10 Fig — The results are displayed for transmission parameters α, β, ϵ and μ. (TIF) [file pcbi.1006697.s027.tif]

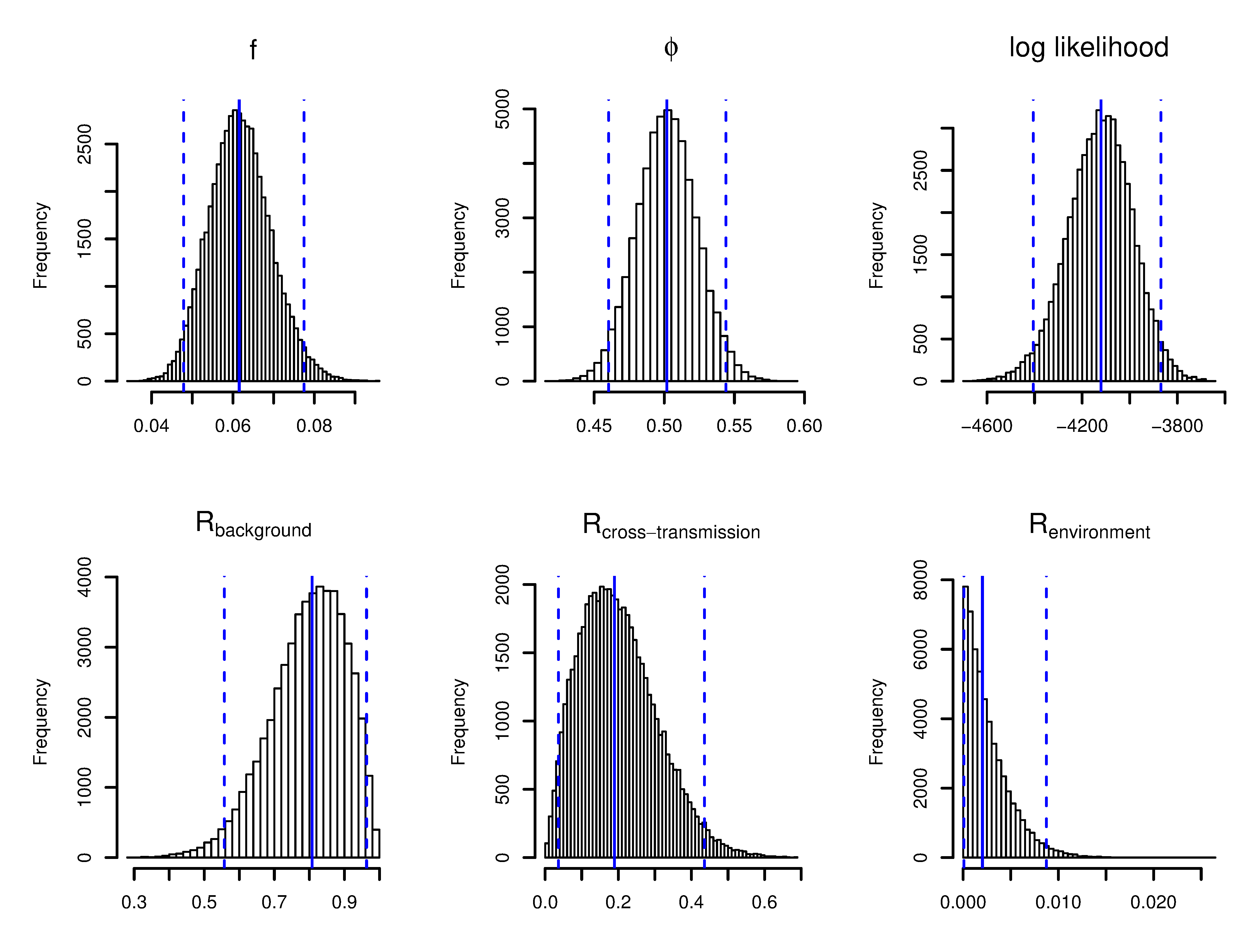

Supplement: S11 Fig — The results are displayed for the importation probability f, sensitivity parameter ϕ, relative contributions Ri, i ∈ {background, cross- transmission, environment} and log-likelihood. (TIF) [file pcbi.1006697.s028.tif]

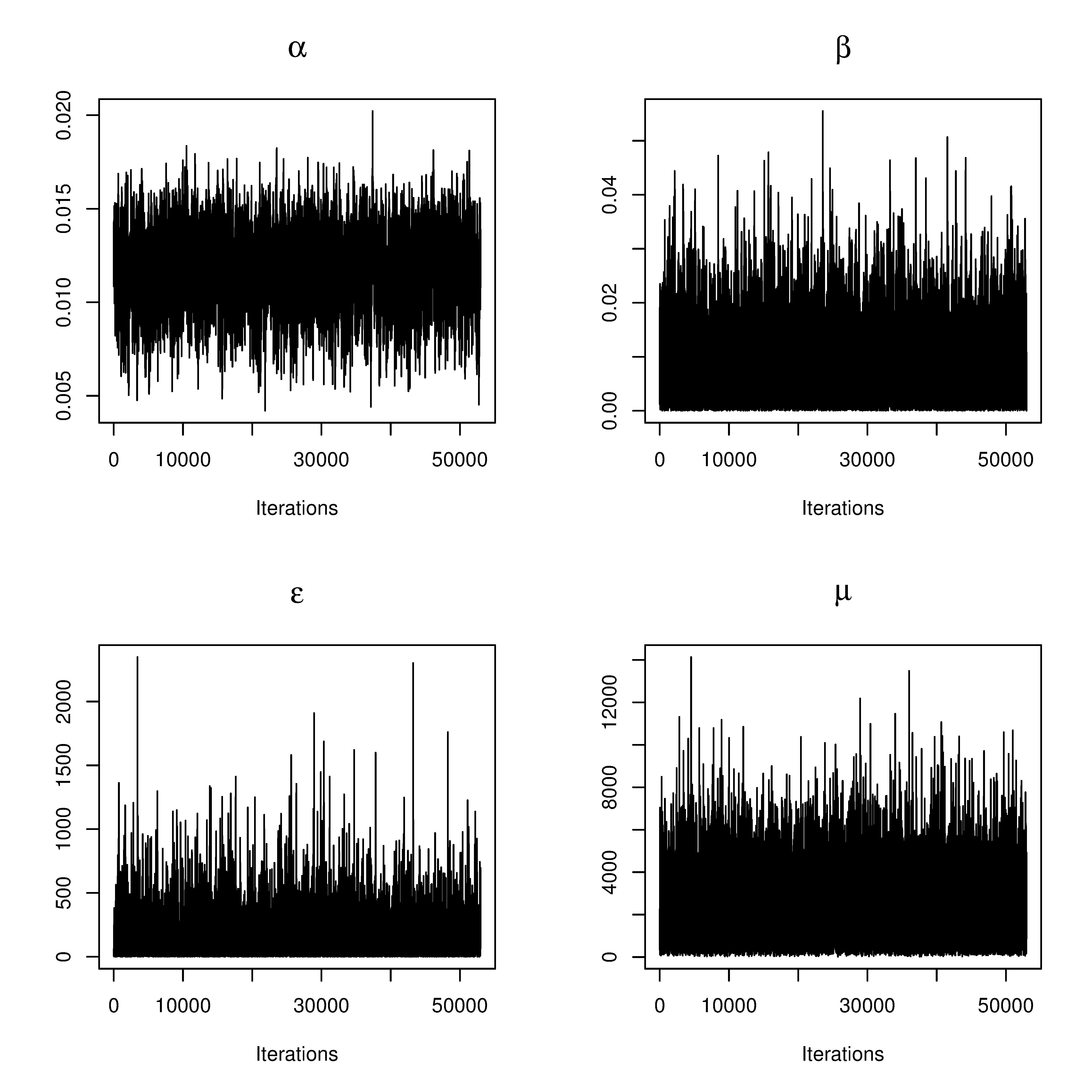

Supplement: S12 Fig — The results are displayed for transmission parameters α, β, ϵ and μ. (TIF) [file pcbi.1006697.s029.tif]

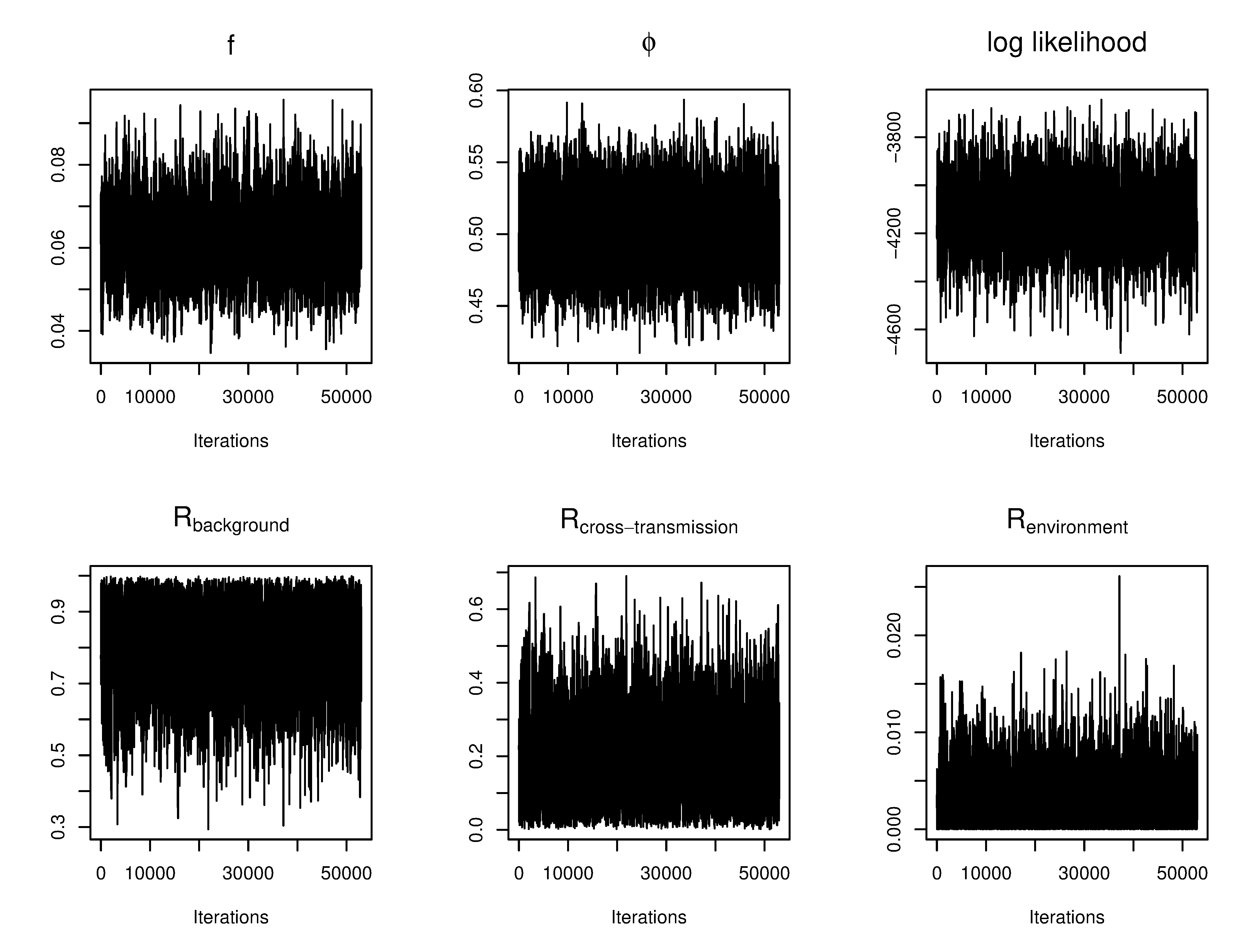

Supplement: S13 Fig — The results are displayed for the importation probability f, sensitivity parameter ϕ, relative contributions Ri, i ∈ {background, cross- transmission, environment} and log-likelihood. (TIF) [file pcbi.1006697.s030.tif]

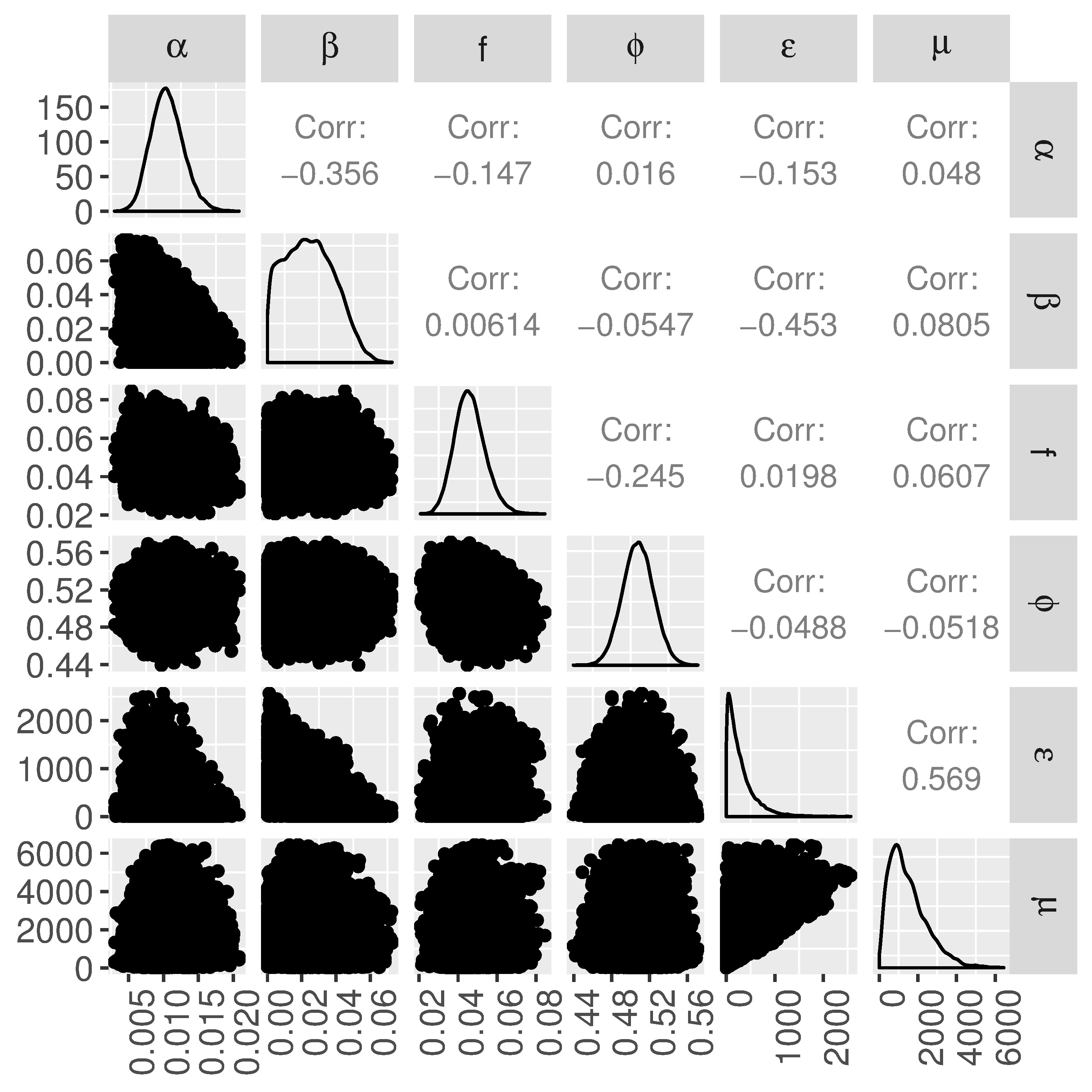

Supplement: S14 Fig — The plots were generated from the data of ICU A before renovation using Exp(0.001) prior and the full model with background, cross-transmission and environmental contamination. (TIF) [file pcbi.1006697.s031.tif]

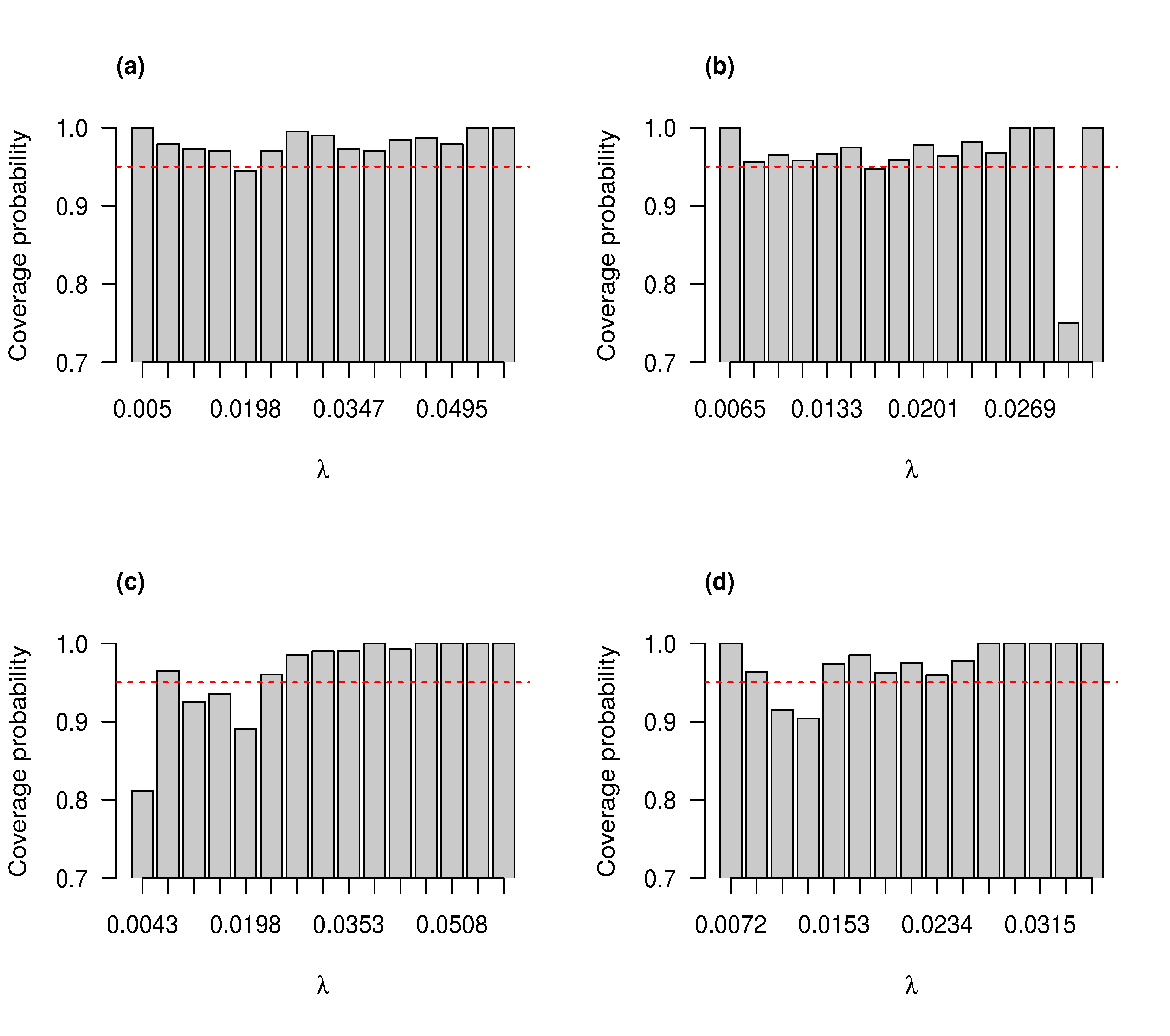

Supplement: S15 Fig — (a)—(b) ICU A before and after renovation, respectively. (c)—(d) ICU B before and after renovation, respectively. (TIF) [file pcbi.1006697.s032.tif]

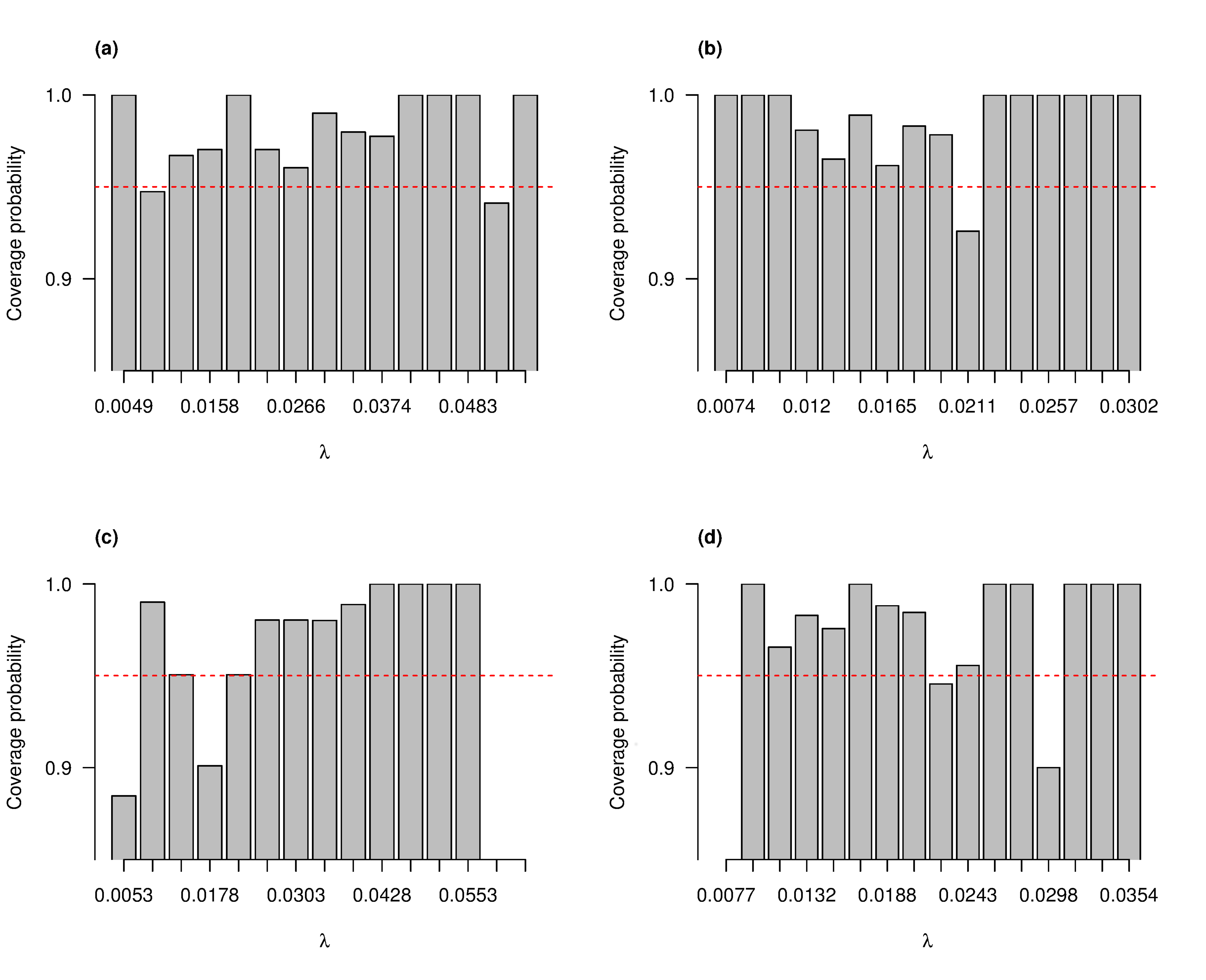

Supplement: S16 Fig — (a)—(b) ICU A before and after renovation, respectively. (c)—(d) ICU B before and after renovation, respectively. (TIF) [file pcbi.1006697.s033.tif]

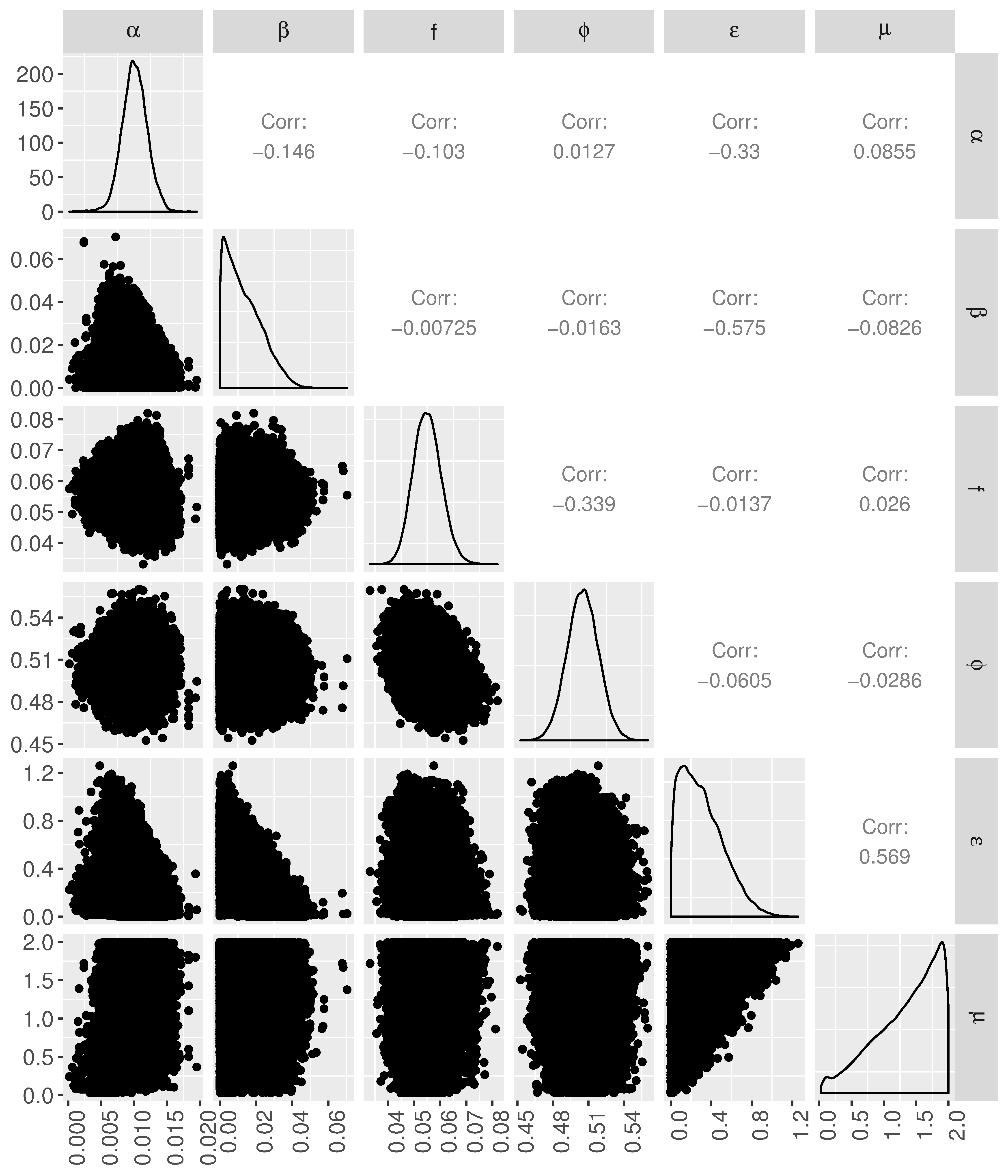

Supplement: S17 Fig — The plots were generated from the data of ICU A using U(0,2) prior and the full model with background, cross-transmission and environmental contamination after discharge. (TIF) [file pcbi.1006697.s034.tif]

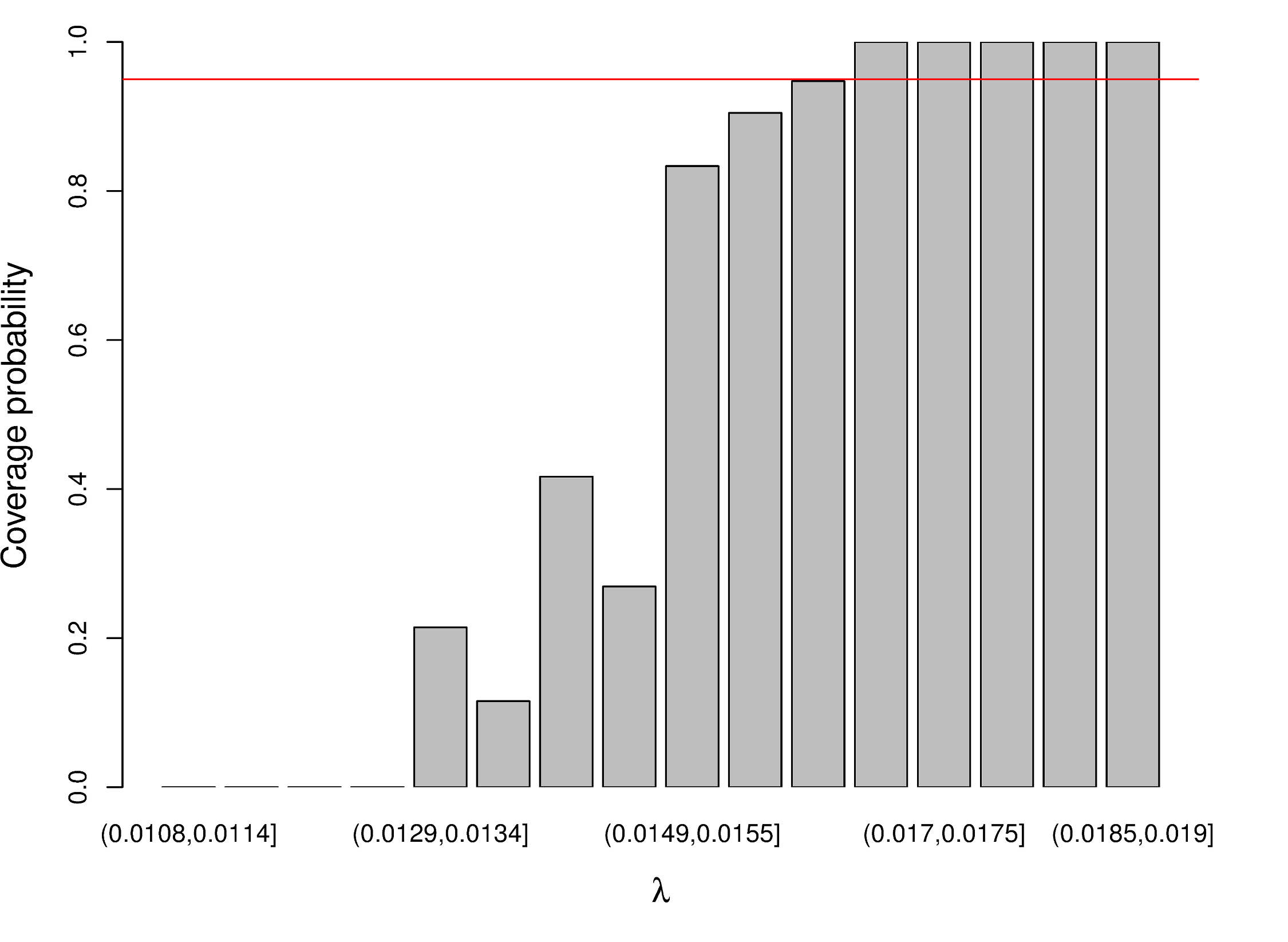

Supplement: S18 Fig — The data was simulated using α = 0.015, β = 0.055, μ = 1/7, ϵ = 0.15, f = 0.05, ϕ = 1. The analysis assumed only one route, i.e. background transmission. The plot shows large discrepancies between the expected and the computed coverage probabilities, pointing to a misspecified model. (TIF) [file pcbi.1006697.s035.tif]
